# Caveolin-1 gene expression provides additional prognostic information combined with PAM50 risk of recurrence (ROR) score in breast cancer

**DOI:** 10.1038/s41598-024-57365-8

**Published:** 2024-03-20

**Authors:** Christopher Godina, Mattias Belting, Johan Vallon-Christersson, Karolin Isaksson, Ana Bosch, Helena Jernström

**Affiliations:** 1 Department of Clinical Sciences Lund, Oncology, Lund University and Skåne University Hospital, Barngatan 4, 221 85 Lund, Sweden; 2 Department of Hematology, Oncology and Radiation Physics, Skåne University Hospital, Skåne, Sweden; 3 Department of Immunology, Genetics and Pathology, Science for Life Laboratory, Uppsala University, Uppsala, Sweden; 4 Department of Clinical Sciences Lund, Surgery, Lund University and Kristianstad Hospital, Kristianstad, Sweden

**Keywords:** Caveolin-1, Breast cancer, Molecular profiling, Prognostic markers, PAM50 ROR, Prognostic markers, Breast cancer, Tumour biomarkers, Cancer metabolism, Cancer microenvironment

## Abstract

Combining information from the tumor microenvironment (TME) with PAM50 Risk of Recurrence (ROR) score could improve breast cancer prognostication. Caveolin-1 (CAV1) is a marker of an active TME. CAV1 is a membrane protein involved in cell signaling, extracellular matrix organization, and tumor-stroma interactions. We sought to investigate *CAV1* gene expression in relation to PAM50 subtypes, ROR score, and their joint prognostic impact. *CAV1* expression was compared between PAM50 subtypes and ROR categories in two cohorts (SCAN-B, n = 5326 and METABRIC, n = 1980). *CAV1* expression was assessed in relation to clinical outcomes using Cox regression and adjusted for clinicopathological predictors. Effect modifications between *CAV1* expression and ROR categories on clinical outcome were investigated using multiplicative and additive two-way interaction analyses. Differential gene expression and gene set enrichment analyses were applied to compare high and low expressing *CAV1* tumors. All samples expressed *CAV1* with the highest expression in the Normal-like subtype. Gene modules consistent with epithelial-mesenchymal transition (EMT), hypoxia, and stromal activation were associated with high *CAV1* expression. *CAV1* expression was inversely associated with ROR category. Interactions between *CAV1* expression and ROR categories were observed in both cohorts. High expressing *CAV1* tumors conferred worse prognosis only within the group classified as ROR high. ROR gave markedly different prognostic information depending on the underlying *CAV1* expression. *CAV1*, a potential mediator between the malignant cells and TME, could be a useful biomarker that enhances and further refines PAM50 ROR risk stratification in patients with ROR high tumors and a potential therapeutic target.

## Introduction

Breast cancer remains a clinical challenge^1^. Despite improvements in care, a significant proportion of breast cancer patients relapse^2,3^. During the past decade, molecular profiling of tumors has been implemented in the clinical setting to improve prognostication and treatment selection^3–6^. One example is the PAM50 Risk of Recurrence (ROR) score classification is now clinically used worldwide for a subgroup of breast cancer. The PAM50 ROR score is validated for postmenopausal breast cancer patients with ER^+^ /HER2^−^ tumors receiving five years of endocrine therapy. The ROR score provides prognostic information and can be used to select patients for adjuvant chemotherapy^4,7,8^. However, further prognostication and treatment prediction refinement is still needed^3^. Additional information may be gained by looking beyond the malignant cells of the tumor, which most molecular risk scores are based on, and incorporating information from the tumor microenvironment (TME).

The TME has gained increasing attention for its role in breast cancer development and treatment response^9,10^. It has become increasingly clear that the tumor depends on its surroundings to be able to grow, survive, and metastasize^9,10^. Currently, there are few prognostic markers derived from the TME and these markers are mostly related to immune cells, such as tumor infiltrating lymphocytes^9–11^. Stromal cells, in particular cancer-associated fibroblasts (CAFs), constitute a significant part of the TME and play a key role in modulating various processes such as epithelial-mesenchymal transition (EMT), hypoxia, and angiogenesis, all important for the development of metastasis^12,13^.

Challenges remain in translating findings related to the TME into relevant biomarkers useful in clinical practice, since the TME is highly heterogenous and comprises several distinct cell types^9,10,14^. Depending on the composition of the TME and its interplay with malignant cells, the TME can either be tumor promoting or suppressing^9,10,12^. An interesting biomarker of an active TME is Caveolin-1 (CAV1). CAV1 is a master regulator of cell signaling and vesicular transport and is located in cholesterol-rich plasma membrane raft domains, known as caveolae^15,16^. CAV1 modulates key pathogenic processes involving the TME, including drug internalization, tumor-stroma interactions, hypoxia response, cellular metabolism, inflammation, and EMT^15–17^.

Furthermore, studies have reported that CAV1 protein expression in stromal cells may serve as a prognostic biomarker in breast cancer^16,18–21^. However, the prognostic impact of CAV1 is both context and localization dependent as previously reported^18,19^. It is unclear how the interplay between CAV1 and clinically used molecular risk scores, such as PAM50 ROR, relates to prognosis. Herein, we investigate the role of *CAV1* gene expression in relation to PAM50 subtypes, ROR scores, and their joint impact on clinical outcome in two large breast cancer cohorts.

## Results

### Relationship with clinicopathological variables

**Figure 1:**
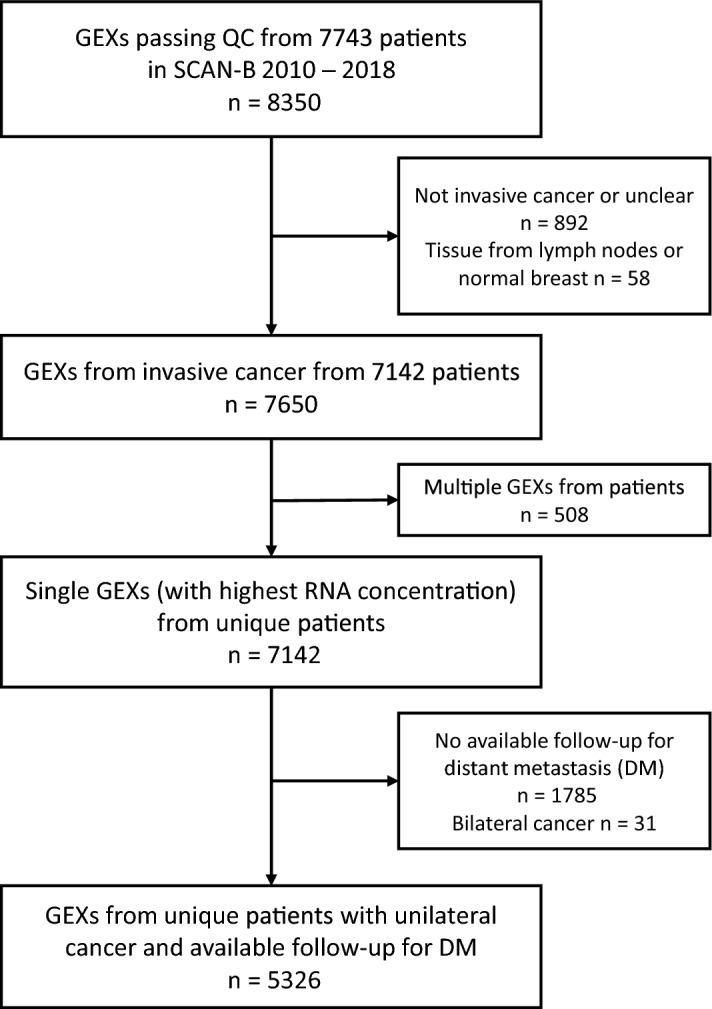
Flowchart of included and excluded patients in SCAN-B.

**Figure 2:**
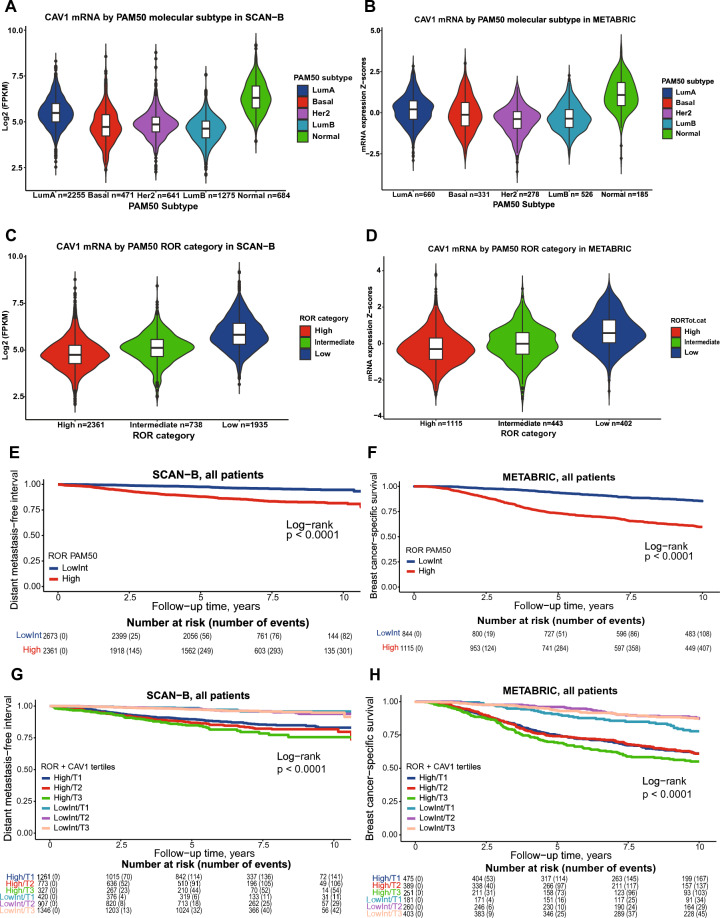
*CAV1* expression by PAM50 and ROR category. *CAV1* expression (continuous) by PAM50 molecular subtype in SCAN-B (**A**) and METABRIC (**B**). *CAV1* expression (continuous) by PAM50 ROR category in SCAN-B (**C**) and METABRIC (**D**). Kaplan–Meier estimates of PAM50 ROR category among all patients in relation to distant metastasis-free interval in SCAN-B (**E**) and breast cancer-specific survival in METABRIC (**F**). Kaplan–Meier estimates of combined ROR category and *CAV1* expression (in tertiles) among all patients in relation to distant metastasis-free interval in SCAN-B (**G**) and breast cancer-specific survival in METABRIC (**H**). The number of patients is indicated at each time-point.

For SCAN-B, 5326 of 7743 patients were assessed in the analysis, Fig. 1. For METABRIC the entire dataset of 1980 patients was used. All samples in both SCAN-B and METABRIC expressed *CAV1*. The distribution of *CAV1* across PAM50 subtypes was similar for both cohorts, with the *CAV1* expression being highest in Normal-like and followed by Luminal A subtype (both *Ps* < 0.001), Fig. 2A,B. Likewise, the correlations between *CAV1* expression and the eight gene modules were similar in both cohorts, showing strong correlations between *CAV1* expression and the Lipid and Stroma modules and ROR category dependent correlation with the Steroid and Immune response modules, Supplementary file 1: Supplementary Fig. 1. Notably, there was an inverse correlation with ROR category in both cohorts (both *r* <  − 0.34 and *P* < 0.001), Fig. 2C,D, Supplementary file 1: Supplementary Fig. 1. Even after adjusting for other predictors of PAM50 ROR category, the highest expression of *CAV1* (T3) was strongly negatively associated with ROR high in both SCAN-B (adjusted OR 0.26 95% CI 0.19–0.35, *P* < 0.001) and METABRIC (adjusted OR 0.32 95% CI 0.21–0.47, *P* < 0.001), Supplementary file 1: Supplementary Fig. 2. *CAV1* gene expression was also negatively correlated with most genes that are a part of the PAM50 ROR, except for *KRT14, KRT15, KRT5,* and *SFRP1*, Supplementary file 1: Supplementary Fig. 2. CAV1 tertiles in relation to clinicopathological factors are presented in Table 1 for SCAN-B and METABRIC.

**Table 1:** Descriptive statistics of CAV1 tertiles in relation to clinicopathological factors in SCAN-B and METABRIC.

	SCAN-B, all patients n = 5326					METABRIC, all patients n = 1980				
	All	Miss-ing	*CAV1* mRNA expression n = 5326			All	Miss-ing	*CAV1* mRNA expression n = 1980		
	patients		Tertile 1	Tertile 2	Tertile 3	patients		Tertile 1	Tertile 2	Tertile 3
	n = 5326		n = 1776	n = 1775	n = 1775	n = 1980		n = 660	n = 660	n = 660
	Number (%)		Number (%)	Number (%)	Number (%)	Number (%)		Number (%)	Number (%)	Number (%)
						Median (IQR)		Median (IQR)	Median (IQR)	Median (IQR)
Age at diagnosis, years	–	0	–	–	–	61.8 (51.4–70.6)	0	64.1 (54.7–72.2)	61.2 (50.8–70.6)	60.1 (50.4–68.5)
–40	270 (5.1)		107 (6.0)	98 (5.5)	65 (3.7)	143 (7.2)		37 (5.6)	47 (7.1)	59 (8.9)
41–50	874 (16.4)		256 (14.4)	368 (20.7)	250 (14.1)	327 (16.5)		89 (13.5)	121 (18.3)	117 (17.7)
51–60	1045 (19.6)		328 (18.5)	353 (19.9)	364 (20.5)	474 (23.9)		142 (21.5)	158 (23.9)	174 (26.4)
61–70	1661 (31.2)		496 (27.9)	530 (29.9)	635 (35.8)	565 (28.5)		201 (30.5)	181 (17.6)	183 (27.7)
71–80	995 (18.7)		358 (20.2)	285 (16.1)	352 (19.8)	367 (18.5)		153 (23.2)	116 (17.6)	98 (14.8)
81–	481 (9.0)		231 (13.0)	141 (7.9)	109 (6.1)	104 (5.3)		38 (5.8)	37 (5.6)	29 (4.4)
Invasive tumor size		166					23			
pT2/3/4 (> 20 mm)	1783 (34.6)		737 (42.8)	550 (31.7)	496 (28.9)	1104 (56.4)		401 (61.3)	383 (58.9)	320 (49.0)
Axillary lymph node involvement		213					0			
pN1/2/3 (any)	1873 (36.6)		674 (39.5)	642 (37.7)	557 (32.7)	937 (47.3)		322 (48.8)	314 (47.6)	301 (45.6)
Main histological type		37					44			
No special type (formerly ductal)	4182 (79.1)		1515 (85.8)	1470 (83.3)	1197 (68.1)	1491 (77.0)		544 (84.0)	515 (79.1)	432 (67.8)
Lobular	732 (13.8)		114 (6.5)	168 (9.5)	450 (25.6)	146 (7.5)		28 (4.3)	35 (5.4)	83 (13.0)
Other or mixed	375 (7.1)		136 (7.7)	127 (7.2)	112 (6.4)	299 (15.4)		76 (11.7)	101 (15.5)	122 (19.2)
Histological grade		382					84			
I	791 (16.0)		131 (7.9)	256 (15.4)	404 (25.0)	169 (8.9)		31 (4.9)	57 (8.9)	81 (12.9)
II	2443 (49.4)		629 (37.8)	854 (51.3)	960 (59.4)	772 (40.7)		226 (35.6)	250 (39.2)	296 (47.4)
III	1710 (34.6)		903 (54.3)	554 (33.3)	253 (15.6)	955 (50.4)		377 (59.5)	330 (51.8)	248 (39.7)
Receptor Status										
ER^+^	4497 (85.2)	49	1388 (78.7)	1520 (86.3)	1589 (90.6)	1506 (76.1)	0	496 (75.2)	489 (74.1)	521 (78.9)
PR^+^	3725 (70.6)	51	1123 (63.7)	1282 (72.8)	1320 (75.3)	1040 (52.5)	0	338 (51.2)	332 (50.3)	370 (56.1)
HER2^+^	702 (13.6)	149	301 (17.2)	257 (15.0)	144 (8.3)	247 (12.5)	0	101 (15.3)	95 (14.4)	51 (7.7)
TNBC	525 (10.4)	64	258 (15.2)	152 (9.1)	115 (6.8)	320 (16.2)	0	113 (17.1)	104 (15.8)	103 (15.6)
Systemic Treatments		39					0			
Endocrine therapy	3901 (78.2)		1315 (75.5)	1401 (80.2)	1393 (79.4)	1216 (61.4)		393 (59.5)	398 (60.3)	425 (64.4)
Chemotherapy	2132 (42.7)		912 (52.4)	755 (43.2)	576 (32.8)	412 (20.8)		127 (19.2)	149 (22.6)	136 (20.6)
Trastuzumab	555 (11.1)		237 (13.6)	217 (12.4)	131 (7.5)	0 (0)		0 (0)	0 (0)	0 (0)
PAM50 Subtypes		0					0			
Luminal A	2555 (42.3)		412 (23.2)	857 (48.3)	986 (55.5)	660 (33.3)		161 (24.4)	228 (34.5)	271 (41.1)
Luminal B	1275 (23.9)		797 (44.9)	397 (22.4)	81 (4.6)	526 (26.6)		239 (36.2)	179 (27.1)	108 (16.4)
Normal-like	684 (12.8)		14 (0.1)	125 (7.0)	545 (30.7)	185 (9.3)		9 (1.4)	34 (5.2)	142 (21.5)
HER2 enriched	641 (12.0)		297 (16.7)	259 (14.6)	85 (4.8)	278 (14.0)		125 (18.9)	119 (18.0)	34 (5.2)
Basal	471 (8.8)		256 (16.7)	137 (7.7)	78 (4.8)	331 (16.7)		126 (19.1)	100 (15.2)	105 (15.9)
PAM50 ROR		292					20			
Low	1935 (38.4)		183 (10.9)	579 (34.5)	1173 (70.1)	402 (20.5)		44 (6.7)	119 (18.3)	239 (36.5)
Intermediate	738 (14.7)		237 (14.1)	328 (19.5)	173 (10.3)	443 (22.6)		137 (20.8)	142 (21.8)	164 (25.1)
High	2361 (46.9)		1261 (75.0)	773 (46.0)	327 (19.5)	1115 (56.9)		475 (72.4)	389 (59.8)	251 (38.4)

### Survival analysis

In SCAN-B, the median follow-up for the 4158 patients still at risk was 5.45 (IQR 5.07–8.15) years. Follow-up was restricted to ten years in METABRIC and all events after ten years were censored. This was done for two reasons; to make METABRIC more comparable to SCAN-B and because the PAM50 ROR score was developed to predict the risk of distant metastasis within 10 years^22^. The median follow-up for the 1089 patients still at risk in METABRIC was 10.0 years (IQR 10.0–10.0). The hazards were proportional for the tertiles for all endpoints.

**Figure 3:**
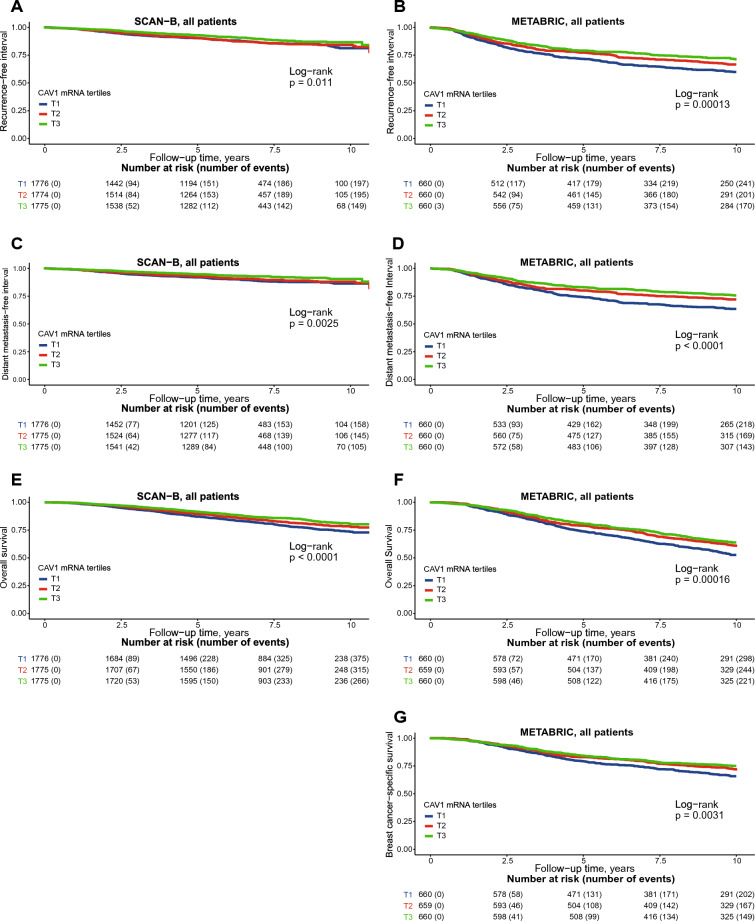
Univariable survival analyses of *CAV1* expression. Kaplan–Meier estimates of *CAV1* expression (in tertiles) among all patients in relation to recurrence-free interval in SCAN-B (**A**) and METABRIC (**B**), distant metastasis-free interval in SCAN-B (**C**) and METABRIC (**D**), overall survival in SCAN-B (**E**) and METABRIC (**F**), and breast cancer-specific survival (**G**). The number of patients is indicated at each time-point.

In the univariable survival analyses of the complete cohorts, patients with ROR high had an increased risk of distant metastasis and breast cancer-specific survival compared to ROR low as expected, Fig. 2E,F. Moreover, the addition of *CAV1* expression further stratified the distant metastasis-risk and breast cancer-specific survival in the univariable models Fig. 2G,H. The highest expression of *CAV1* (T3) was associated with lower risk of recurrence, HR 0.74 (95% CI 0.60–0.92) in SCAN-B and HR 0.66 (95% CI 0.54–0.80) in METABRIC; lower risk of distant metastasis, HR 0.65 (95% CI 0.51–0.84) in SCAN-B and HR 0.61 (95% CI 0.50–0.76) in METABRIC; and lower risk of death, HR 0.68 (95% CI 0.58–0.80) in SCAN-B and HR 0.70 (95% CI 0.59–0.84) in METABRIC, Fig. 3. The highest expression of *CAV1* (T3) also conferred a lower risk of breast cancer-related death HR 0.70 (95% CI 0.57–0.87) in METABRIC, Fig. 3.

In the multivariable analyses, the highest expression of *CAV1* (T3) in SCAN-B instead conferred an increased risk of recurrence and distant metastasis but not death, Fig. 2. and Supplementary file 1: Supplementary Fig. 3. *CAV1* tertiles were therefore adjusted for each variable used in the multivariable model, one at a time to see which variable affected the hazard ratio the most, which was the ROR category. Subsequently, interaction analyses were performed between ROR category and *CAV1* tertiles on RFI and DMFI, revealing significant additive interactions and effect modifications of both ROR category on *CAV1* and vice versa, Table 2. In SCAN-B, when stratifying by ROR category, the highest expression of *CAV1* (T3) conferred increased risk of recurrence adjusted HR 1.57 (95% CI 1.10–2.24) and distant metastasis adjusted HR 1.60 (95% CI 1.08–2.37) only in patients with tumors classified as ROR High but not in ROR Low/Intermediate tumors, Supplementary file 1: Supplementary Fig. 4 and 5.

**Table 2:** Full report of interactions between ROR High and *CAV1* T3 on DMFI.

	*CAV1* T1	*CAV1* T3	Effect of *CAV1* T3 within each stratum of ROR
	HR (95% CI)	HR (95% CI)	HR (95% CI)
Interaction between ROR High and *CAV1* T3 on DMFI in SCAN-B			
ROR Low/Intermediate	1	1.04 (0.52, 2.07)	1.04 (0.52, 2.07)
	Reference	*P* > 0.3	*P* > 0.3
ROR High	1.90 (0.97, 3.72)	3.11 (1.55, 6.25)	1.64 (1.12, 2.41)
	*P* = 0.063	*P* = 0.001	*P* = 0.011
Effect of ROR High within each stratum of *CAV1*	1.90 (0.97, 3.72)	2.99 (1.84, 4.86)	
	*P* = 0.063	*P* < 0.001	
Multiplicative scale	1.58 (0.74, 3.38)		
	*P* = 0.24		
RERI	1.18 (0.10, 2.26)		
	*P* = 0.016		
AP	0.38 (0.07, 0.69)		
	*P* = 0.009		
Interaction between ROR High and *CAV1* T3 on DMFI in METABRIC			
ROR Low/Intermediate	1	0.51 (0.32, 0.81)	0.51 (0.32, 0.81)
	Reference	*P* = 0.004	*P* = 0.004
ROR High	1.02 (0.68, 1.54)	1.07 (0.69, 1.66)	1.05 (0.81, 1.37)
	P > 0.3	P > 0.3	P > 0.3
Effect of ROR High within each stratum of *CAV1*	1.02 (0.68, 1.54)	2.10 (1.39, 3.18)	
	P > 0.3	P < 0.001	
Multiplicative scale	2.06 (1.23, 3.45)		
	*P* = 0.006		
RERI	0.54 (0.20, 0.89)		
	*P* = 0.001		
AP	0.50 (0.11, 0.90)		
	*P* = 0.006		

*RERI* Relative risk due to interaction.

*AP* Attributable Portion.

The distribution of ROR categories in the two cohorts differed with a larger proportion of tumors classified as ROR High in METABRIC than in SCAN-B. Considering the interactions between ROR categories and *CAV1* expression in SCAN-B, interaction analyses between ROR categories and *CAV1* tertiles for all four endpoints were performed to investigate if potential effect modifications previously seen in SCAN-B was the underlying reason for the discrepant findings on prognosis in METABRIC. (In METABRIC, associations between *CAV1* tertiles and either one of the endpoints were not statistically significant in the multivariable analysis, Supplementary file 1: Supplementary Fig. 3.) Subsequently, it became clear that there were significant multiplicate and additive interactions between *CAV1* T3 and ROR category on DMFI, OS, and BCSS in METABRIC, Table 2 and Supplementary file 2: Supplementary Table 1, 2, and 3. Similarly, there were effect modifications of both ROR category on *CAV1* and vice versa concerning prognosis, Table 2 and Supplementary file 1: Supplementary Table 1, 2, and 3. Interestingly, in both SCAN-B and METABRIC, *CAV1* tertiles could identify tumors where the predictive potential of ROR was the highest, Table 2 and Supplementary file 1: Supplementary Table 1, 2, and 3. Similar to DMFI in SCAN-B, when stratifying by ROR category in METABRIC, the highest expression of *CAV1* (T3) conferred borderline increased risk of breast cancer-specific death adjusted HR 1.24 (95% CI 0.95–1.62) only in patients with tumors classified as ROR High but not in ROR Low/Intermediate tumors, Table 2, Fig. 2, Supplementary file 1: Supplementary Fig. 6 and 7. The additive interaction was present in both cohorts but was stronger in SCAN-B RERI 1.18 (95% CI 0.10–2.26, P = 0.016) than in METABRIC RERI 0.54 (95% CI 0.20–0.89,* P* = 0.001) Table 2. For METABRIC, the additive interaction was even stronger when breast cancer-specific survival was used as endpoint RERI 0.77 (95% CI 0.43–1.12, *P* < 0.001), Fig. 2H. Furthermore, *CAV1* tertiles could also delineate in which group the ROR category was prognostic. In the *CAV1* T1 tumors, the ROR category did not predict risk of distant metastasis, Supplementary file 1: Supplementary Fig. 8. In *CAV1* T2 tumors, the ROR category predicted distant metastasis risk in SCAN-B but not METABRIC Supplementary file 1: Supplementary Fig. 9. For the *CAV1* T3 tumors, ROR category was strongly associated with distant metastasis risk in both SCAN-B and METABRIC Supplementary file 1: Supplementary Fig. 10.

### DGE and GSEA analysis for CAV1 Tertile 3 vs Tertile 1

To elucidate potential biological explanations behind the differential impact of *CAV1* according to ROR category, DGE analyses were performed separately in ROR categories for tumors with the highest (T3) versus the lowest (T1) *CAV1* expression.

In ROR high tumors, a total of 223 genes were found to be upregulated in high expressing (T3) *vs* low expressing (T1) *CAV1* tumors, and no genes were downregulated, Supplementary file 2: Supplementary Table 4. Notably, several other genes coding for proteins involved in caveolae formation, *e.g. CAV2* and *CAVIN2* were higher expressed in ROR high/*CAV1* high tumors, supporting a potential association with caveolae abundance. In ROR Low/Intermediate tumors, 450 genes were upregulated, and 18 genes were downregulated in high expressing (T3) *vs* low expressing (T1) *CAV1* tumors, Supplementary file 2: Supplementary Table 5. In both ROR categories, genes related to stromal activation, EMT, CAFs and adipogenesis (*SOX10, STAC2, FGF2, PTGFR, IGF1, IGF2, GDF10, ADAM33, CD36, PLIN4, PLIN1, MME, PENK,* among others) were upregulated, Supplementary file 2: Supplementary Table 4 and 5. Only in ROR low/intermediate tumors were some potential tumor suppressor genes down-regulated (*EEF1A2, GRM4, ROBO2 CHGB, CEACAM5,* among others), Supplementary file 2: Supplementary Table 5.

**Figure 4:**
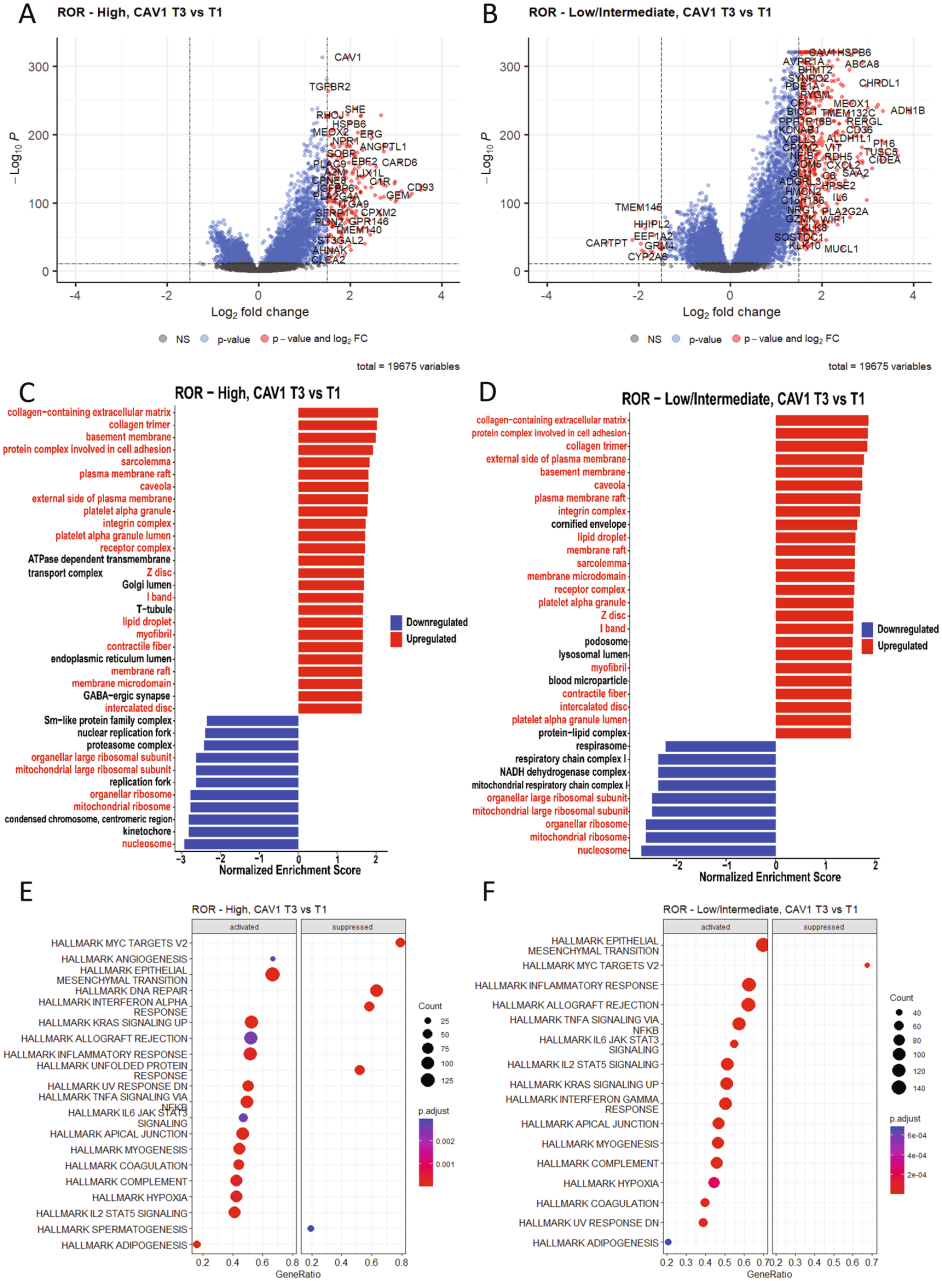
Molecular analyses of *CAV1* expression. Volcano plot showing significant up- and downregulated genes (red) in high expressing (T3) in relation to low expressing (T1) *CAV1* separately by ROR High category (**A**) and ROR Low/Intermediate category (**B**). Gene Set Enrichment Analysis (GO categories) of genes ranked by fold change (log2FC) and p-value < 0.05**,** up- and downregulated genes (red) in high expressing (T3) in relation to low expressing (T1) *CAV1* tumors separately by ROR High category (**C**) and ROR Low/Intermediate category (**D**). Categories found enriched in both subgroup analyses are indicated by red text. Dot plot showing activated and suppressed Hallmark Signatures in high expressing (T3) in relation to low expressing (T1) *CAV1* tumors separately by ROR High category (**E**) and ROR Low/Intermediate category (**F**).

Significantly enriched gene sets in high expressing (T3) *CAV1* tumors in both ROR categories included EMT, TGF-β signaling, fatty acid metabolism, hypoxia, myogenesis, angiogenesis, xenobiotic metabolism among others, Fig. 4 and Supplementary file 2: Supplementary Tables 6 and 7. In low expressing (T1) *CAV1* tumors regardless of ROR category, the MYC targets gene set was enriched, Fig. 4 and Supplementary file 2: Supplementary Tables 6 and 7. The main differences in gene set enrichment between high expressing (T3) *CAV1* tumors and low expressing (T1) *CAV1* tumors were related to immune response. Among high expressing (T3) *CAV1* tumors, interferon-α response and complement hallmarks were enriched only in ROR high, while interferon-γ response hallmark were enriched only in ROR low/intermediate, Fig. 4 and Supplementary file 2: Supplementary Tables 6 and 7. Similar patterns were seen regarding GO terms, Supplementary file 2: Supplementary Tables 8 and 9.

## Discussion

Herein, we report that high *CAV1* gene expression conferred an especially poor prognosis in patients whose tumors were classified as ROR high. In addition, ROR gave markedly different prognostic information depending on the underlying *CAV1* expression, even after taking PAM50 subtype, other clinical predictors, and treatments into account. To our knowledge, this is the first study to examine the *CAV1* mRNA gene expression in relation to molecular subtypes and prognosis in large breast cancer cohorts.

Moreover, *CAV1* expression was associated with extracellular matrix remodeling, EMT myogenesis, hypoxia, angiogenesis, and stromal activation in both ROR High and Low/Intermediate classified tumors, as corroborated by both GSEA results and their correlations with the stromal gene module. It is known that CAV1 can remodel the extra cellular matrix through activation of stromal cells, elongating and facilitating invasion and metastasis^23^. Functionally, alterations in caveolae in stromal cells of the TME promote paracrine tumor growth via TGFβ, which activates EMT and myofibroblast differentiation, favoring tumor growth and metastasis^24,25^. EMT and myogenesis are markers of increased cell motility and loss of adhesion, both required for metastasis^26^.

Furthermore, CAV1 is linked to angiogenesis, endothelial permeability, and vascular endothelial growth factor (VEGF) response, which is required for tumor survival and the ability to enter the circulation. However, the exact role of CAV1 is unclear^27^. Hypoxia and angiogenesis are interlinked, and CAV1 is a direct transcriptional target of hypoxia-inducible factors 1α and 2α (HIF1α and 2α) that lead to increased dimerization and phosphorylation of the epidermal growth factor receptor (EGFR) conferring enhancing proliferative, migratory, and invasive capacities of malignant cells^28^. Further, hypoxia induces metabolic reprogramming in the tumor and CAV1 alterations confer a shift from mitochondrial respiration to tumor promoting aerobic glycolysis through attenuation of MYC expression^29^, corroborated by our data as seen in the downregulation of MYC response.

So far, the role of CAV1 in the immunomodulatory properties of the TME remains unexplored and further studies are warranted. The types of immune signals enriched in high expressing *CAV1* tumors were dependent on ROR category and activation of immune response appeared higher in ROR low/intermediate tumors. The stromal microenvironment has immunomodulatory functions and can inhibit immune cells and decrease their efficacy in targeting and killing malignant cells, through regulation of extravasation and local immune cell replication^9–11^.

Our findings and existing literature suggest that CAV1 promotes metastasis and relapse through several critical pathways regardless of genomic risk classification. CAV1 can be considered as an essential protein that regulates paracrine signaling and the interplay between the malignant cells and TME. However, *CAV1* expression only yielded additional prognostic information in tumors considered ROR high. A potential explanation for this finding might be that malignant cells that already acquired the intrinsic potential to metastasize still need an active and tumor promoting environment to do so. It might explain why high *CAV1* expression in tumors identified patients for whom the ROR score provided most prognostic information. In contrast, the ROR score only gave little prognostic information in patients whose tumors had low *CAV1* expression. It has been hypothesized that both an active tumor promoting TME, and oncogenic intrinsic features of malignant cells are needed for the tumor to be able to metastasize^9,10^, which is in line with our findings.

Our study examined mRNA rather than protein levels. In addition to mRNA expression, protein levels are also affected by translation, post-translational modifications, and regulation of the rate of protein decay^30^. The global correlation between mRNA and protein is expected to be high^30^. We have previously reported a correlation of R_*s*_ = 0.47 for CAV1^18^. Consequently, the present study results must be interpreted in the context of the biological phenotype related to high *CAV1* mRNA expression.

The standard treatment regimens differ between SCAN-B and METABRIC^31–35^ mainly due to samples being collected during different time periods^31,34,35^. Therefore, there is a large discrepancy in the type of treatments between the older METABRIC and the contemporary SCAN-B cohort. Differences in treatments could explain why the results regarding prognosis were not fully replicated. CAV1 has been shown to modulate treatment efficacy of chemotherapy (including epirubicin and taxanes) and trastuzumab in breast cancer and other cancers^36–39^. These treatments were rarely or not at all used in METABRIC. Our findings in SCAN-B may partly be explained by how CAV1 modulates these treatments since patients with ROR high tumors are more likely to receive chemotherapy and trastuzumab, where treatment efficacy partly depends on *CAV1* expression. Unfortunately, a lack of more detailed information on treatments makes it hard to evaluate the role of *CAV1* expression in response to specific treatments in our study. Assessment of *CAV1* expression in tumor samples from previous randomized clinical trials is warranted to confirm whether *CAV1* expression may further refine ROR score prediction.

It should be mentioned that the PAM50 ROR score is used clinically for risk prediction in postmenopausal patients with ER^+^ /HER2^−^ tumors to identify patients where the recurrence risk is low enough to omit chemotherapy^4,7,8^. However, studies have shown that in ER negative disease, (ER^−^ /HER2^+^ and TNBC), both PAM50 subtype and ROR score can predict neoadjuvant treatment response^40–43^. Further, in TNBC disease, PAM50 subtype categorization could predict sensitivity to taxanes and capecitabine treatment^44^. Similarly, PAM50 subtype and ROR score were shown to be prognostic in HER2^+^ disease^45^. Therefore, PAM50 ROR could play a role in more than one clinical subgroup of breast cancer, and we believe it is of interest to study the ROR score in a broader context. Our data indicate that *CAV1* expression could identify tumors where ROR score was prognostic, potentially broadening the applicability of the PAM50 ROR score, beyond the subgroup of ER^+^ /HER2^−^ tumors.

Beyond the type of treatment, there could be several other reasons why the results regarding prognosis were not precisely replicated in the METABRIC. First, the METABRIC is a smaller cohort, hence, sample sizes in the tests are smaller and potential survival associations may not be as readily detectable. METABRIC also consists of more advanced tumors and is not population-based^46^, which is reflected by differences between the cohorts in the distribution of clinicopathological variables, including ROR category. Since nodal status is the key factor for determining ROR category and nodal status was substantially higher in METABRIC than in SCAN-B, this fact may in part explain why the prognostic impact of *CAV1* gene expression differed somewhat between the cohorts. The underlying risk of recurrence and death in METABRIC is considerably higher than in SCAN-B, making direct comparisons regarding prognosis difficult^46^. The derived *CAV1* tertile classifications are relative to a population and not based on absolute cut-offs for each tumor. The tertile cut-offs were applied separately for each cohort, meaning that some tumors would be reclassified if a unform cut-off had been applied. One might expect that relatively more tumors in METABRIC would have be classified as low *CAV1* expressing due to the inverse association between *CAV1* expression and tumor aggressiveness^46^.

It should be noted that the gene expression data is derived from bulk tumors, which reflects the averaged gene expression across thousands of cells and different cell types^47^. Therefore, it was not possible to definitively infer which cell types CAV1 was located in^47^ and, subsequently, the role CAV1 plays in these cell types in the context of breast cancer. Unfortunately, to date, no available assays can apply single-cell resolution for large-scale cohorts such as SCAN-B and METABRIC to evaluate prognostic biomarkers.

Nonetheless, the population-based contemporary SCAN-B cohort offers unique advantages, which lies in the large-scale RNAseq analysis of consecutively enrolled breast cancers^32,33,48^. To our knowledge, this is the largest cohort of its kind to date. Due to a rigorous population-based approach with consistently high inclusion rates because of seamless integration of patient enrollment and tissue sampling incorporated into routine clinical practice, the study cohort can be considered representative of the general patient demographics in the catchment area^32,33,48^. Therefore, SCAN-B allows for the evaluation of biomarkers in a contemporary real-world setting. Further, most findings were confirmed, showing stable associations of *CAV1* expression with clinicopathological factors and tumor biology, consistent with the literature. In both cohorts, similar additive interactions with ROR regarding clinical outcome were shown as well as the underlying *CAV1* expression being able to markedly change the prognostic information yielded by PAM50 ROR.

In conclusion, our findings indicate that high *CAV1* gene expression is associated with a particularly poor prognosis in patients with ROR high tumors. As CAV1 can mediate between malignant cells and the TME it may also be a promising therapeutic target. The underlying *CAV1* expression markedly modified the prognostic information provided by PAM50 ROR. We have shown in two independent datasets that PAM50 ROR was only prognostic in tumors with high *CAV1* expression. Thus, *CAV1* expression could be a useful biomarker that may enhance and further refine PAM50 ROR risk stratification for patients with ROR high tumors.

## Materials and methods

### SCAN-B

The Swedish Cancerome Analysis Network—Breast (SCAN-B: ClinicalTrials.gov ID NCT02306096) is an ongoing population-based study that have enrolled breast cancer patients at seven hospitals in South Sweden and two additional hospitals (Uppsala and Jönköping)^32,33^. The enrollment of patients is integrated in clinical routine^33^ and all patients with newly diagnosed or suspected breast cancer are invited to participate. The Swedish National Quality Registry for Breast Cancer is used for collection of clinicopathological data, treatment information, and follow-up^32,33,48^.

Sample collection followed established SCAN-B procedures and protocols^32,33^. In brief, the remaining fresh collected tumor samples from surgical specimens were preserved in RNAlater (Qiagen, Hilden, Germany). Core needle biopsies were taken before neoadjuvant treatment and preserved in RNAlater. Gene expression profiling of the tumors was performed by massive parallel paired-end sequencing of mRNA (RNA-seq) using a custom SCAN-B workflow^32,48^. Details on library preparation, quality control, the analysis pipeline, and software used are described elsewhere^32,33,48^.

All clinicopathological data and gene expression data for SCAN-B patients used here were downloaded from the Supplementary Information and Data from Staaf et al.^48^. Expression levels were expressed in fragments per kilobase of exon per million mapped reads (FPKM) in an expression matrix^48^. To all FPKM data an offset of + 0.1 was added, and then the data was log2 transformed.

Patients were enrolled between September 1, 2010, and May 31, 2018, and followed until November 2021^48^. From the beginning 7743 patients were included with a total of 8350 gene expression profiles (GEXs), as previously described^48^. After exclusion of GEXs from noninvasive cancer or lymph nodes, a total of 7142 patients remained with 7650 GEXs in the current study. In case multiple gene expression profiles from a single tumor passed quality control, the profile with the highest RNA concentration measured by NanoDrop spectrophotometry was chosen, as previously described^48^.This procedure left one GEX per patient for analysis. Further, patients with bilateral cancer or no available follow-up for distant metastasis were excluded, Fig. 1. After exclusions, GEX profiles from a total of 5326 patients were available for analysis. Information on PAM50 subtype and ROR category was obtained from Staaf et al*.*^48^ who assigned these categories using single sample predictors^48^.

### Metabric

Molecular Taxonomy of Breast Cancer International Consortium (METABRIC) is collection of clinically annotated primary fresh-frozen breast cancer specimens from five tumor banks in the UK and Canada^34^. The patients were diagnosed with non-metastatic breast cancer between 1977 and 2005^34^. Manual curation and basic quality control of the clinicopathological data including treatment information was performed^31^. None of the HER2^+^ patients received trastuzumab. For a subset of 1980 patients, known as the METABRIC molecular dataset, gene-expression data from microarrays is available^31,34,35^. Details on sample handling, gene expression profiling and workflow are described elsewhere^34,35^. The METABRIC molecular dataset was downloaded from https://www.cbioportal.org/study/summary?id=brca_metabric and corresponding clinical data from Rueda et al.^31^. The *genefu* package^49^ was used to assign PAM50 subtype using nearest centroid correlation^22^ and calculate the PAM50 ROR score based on centroid correlations, tumor size and proliferation score according to the ROR equation with nodal status dependent cut-offs to assign categories, as described^7,50,51^.

In both SCAN-B and METABRIC, eight gene expression modules representing different biological functions in breast cancer were calculated as previously described^52^.

### Statistical analysis

Differences in *CAV1* mRNA expression depending on PAM50 subtype were evaluated using Kruskal–Wallis test. Correlations between *CAV1* expression, ROR category and the eight gene modules^52^ were assessed using Pearson’s correlation (*r*). Pearson’s correlation was also used to assess correlations between *CAV1* mRNA expression and mRNA expression of the PAM50 genes. Logistic regression was used to test whether *CAV1* mRNA expression was independently associated with ROR category after adjusting for potential confounders (age at diagnosis, axillary lymph node status (pN1/2/3), tumor size (pT2/3/4), Grade (III vs I or II), ER^+^, PR^+^, HER2^+^, PAM50 subtype (Luminal A as reference), and (neo)adjuvant treatments.

Endpoints used for survival analysis were recurrence-free interval (RFI), distant metastasis-free interval (DMFI), and overall survival (OS) for both SCAN-B and METABRIC, as previously defined^31,34,48^. Breast cancer-specific survival (BCSS) was used as additional endpoint for METABRIC^31,34^.

For the survival analysis, log2 transformed *CAV1* mRNA expression were categorized into tertiles, tertile 1 (T1), tertile 2 (T2), and tertile 3 (T3) to allow for non-linear effects. The lowest expression of *CAV1* (T1) was used as reference. For survival analyses, the R packages ‘survival’ and ‘survminer’ were used.

Univariable survival analyses were performed using the Kaplan–Meier method and the Log-rank test. Cox proportional hazards models were used to obtain crude and adjusted Hazard ratios (HRs) with 95% confidence intervals (CI). The multivariable models were adjusted for age (binned in 5-year intervals for SCAN-B or continuous for METABRIC), tumor characteristics; axillary lymph node status (pN1/2/3), tumor size (pT2/3/4), Grade (III vs I or II), ER^+^, PR^+^, HER2^+^, PAM50 subtype (Luminal A as reference), PAM50 ROR category (High vs Low/Intermediate); and (neo)adjuvant treatments (endocrine treatment and chemotherapy for both SCAN-B and METABRIC and trastuzmab for SCAN-B only).

Schoenfeld’s residuals were used to test and graphically examine the proportional hazard assumption for the CAV1 tertiles in the adjusted model. To investigate effect modifications between the *CAV1* tertiles and PAM50 ROR category, two-way interaction analyses on multiplicative and additive scales were performed in the multivariable model using the ‘interactionR’ package^53^.

Differential gene expression (DGE) analysis was conducted in SCAN-B using the ‘Limma-Voom’ package^54^ to find differentially expressed genes (DEGs) between the highest tertile (T3) and the lowest tertile (T1) of *CAV1* expression. The criteria used to define DEGs is a false discovery rate (FDR) of ≤ 0.05 and log2 fold change (log2FC) ≥ 1.5 for up-regulated genes and log2FC ≤  − 1.5 for down-regulated genes. To correct for batch effects, batch was included in the Limma models. Gene set enrichment analysis (GSEA) was performed in ‘clusterprofiler’^55^ to find the statistically significant, concordant gene sets that differed between the highest tertile (T3) and the lowest tertile (T1) of *CAV1* expression. Gene sets were grouped according to Gene Ontology (GO) and Hallmark Signature annotations^56,57^.

All statistical analyses were conducted in R version 4.2.2. *P*-values < 0.05 was considered statistically significant. All *P*-values were two-tailed. This study followed the Reporting Recommendations for Tumor Marker Prognostic Studies (REMARK) criteria^58^.

### Ethics approval and consent to participate

Ethical approvals for the cohorts studied (SCAN-B and METABRIC) were obtained in relation to the primary projects and publications^31–35,48^. The SCAN-B study was approved by the Lund University ethics committee^32,33,48^. The METABRIC study was approved by the ethics committees at the University of Cambridge and the British Columbia Cancer Research Centre ^31,34,35^. All participants signed written informed consent. No separate approval was obtained for this specific study since it is based on previously published data. The study was conducted in accordance with the ethical principles of the Declaration of Helsinki.

### Supplementary Information

Supplementary Information 1.

Supplementary Information 2.

## Data Availability

RNA-sequencing-based gene expression data for the SCAN-B cohort is a publicly accessible dataset from Staaf et al.^48^ available at Mendeley Data. Microarray-based gene-expression data for METABRIC is publicly available from Curtis et al.^34^ and Pereira et al.^35^ at cBioPortal and clinical data is a publicly accessible dataset from Rueda et al.^31^.

## Abbreviations

BCSS: Breast cancer-specific survival
CAF: Cancer-associated fibroblast
CAV1: Caveolin-1
DEGs: Differentially expressed genes
DGE: Differential gene expression
DMFI: Distant metastasis-free interval
EGFR: Epidermal growth factor receptor
ER: Estrogen receptor
EMT: Epithelial-mesenchymal transition
FC: Fold change
FDR: False discovery rate
FPKM: Fragments per kilobase of exon per million mapped reads
GEX: Gene expression profile
GO: Gene ontology
GSEA: Gene set enrichment analysis
HER2: Human epidermal growth factor receptor 2
HIF1α: Hypoxia-inducible factor 1 α
HIF2α: Hypoxia-inducible factor 2 α
HR: Hazard ratio
IHC: Immunohistochemistry
OS: Overall survival
METABRIC: Molecular taxonomy of breast cancer international Consortium
PR: Progesterone receptor
RFI: Recurrence-free interval
ROR: Risk of recurrence
RNA-seq: Massive parallel paired-end sequencing of mRNA
SCAN-B: Swedish cancerome atlas network-Breast
TGFβ: Transforming growth factor-beta
TME: Tumor microenvironment
TNBC: Triple-negative breast cancer
VEGF: Vascular endothelial growth factor
