# Supplementary material for: Caveolin-1 gene expression provides additional prognostic information combined with PAM50 risk of recurrence (ROR) score in breast cancer
Source: Sci Rep. 2024 Mar 20;14:6675. doi: 10.1038/s41598-024-57365-8 (PMC10954762; doi:10.1038/s41598-024-57365-8)

Caveolin-1 gene expression provides additional prognostic information combined with PAM50 Risk of Recurrence (ROR) score in breast cancer

Christopher Godina[1], Mattias Belting[1,2,3], Johan Vallon-Christersson[1], Karolin Isaksson[4], Ana Bosch[1,2], Helena Jernström[1].

1 Division of Oncology, Department of Clinical Sciences in Lund, Lund University and Skåne University Hospital, Barngatan 4, SE-221 85 Lund, Sweden

2 Department of Hematology, Oncology and Radiation Physics, Skåne University Hospital, Sweden

3 Department of Immunology, Genetics and Pathology, Science for Life Laboratory, Uppsala University, Uppsala, Sweden

4 Division of Surgery, Department of Clinical Sciences in Lund, Lund University and Kristianstad Hospital, Kristianstad, Sweden

Table of Contents

Supplementary Figure Legends……………..…………………………………………………...2-5

Supplementary Table 1…………...……………………………………………………………6

Supplementary Table 2…………...……………………………………………………………7

Supplementary Table 3…………...……………………………………………………………8

Supplementary Figure 1………………...…………………………………………………...9

Supplementary Figure 2……………………………………………………………………..10

Supplementary Figure 3……………………………………………………………………..11

Supplementary Figure 4……………………………………………………………………..12

Supplementary Figure 5……………………………………………………………………..13

Supplementary Figure 6……………………………………………………………………..14

Supplementary Figure 7……………………………………………………………………..15

Supplementary Figure 8……………………………………………………………………..16

Supplementary Figure 9……………………………………………………………………..17

Supplementary Figure 10…………………………………………………………...……..18

Supplementary figure legends

Supplementary Fig. 1. Correlation between *CAV1* expression and different biological processes

Pearson correlations of *CAV1* gene expression and the eight gene modules (Stroma, Lipid, Immune Response, Mitotic Checkpoint, Mitotic Progression, Basal, Early Response, Steroid Response) among all patients in SCAN-B **(A)** and METABRIC **(B)**, among patients whose tumors were classified as ROR High in SCAN-B **(C)** and METABRIC **(D)**, and among patients whose tumors were classified as ROR Low/Intermediate in SCAN-B **(E)** and METABRIC **(F)**. All the Pearson correlations of *CAV1* gene expression and the eight gene modules in SCAN-B **(G)** and METABRIC **(H)**,

Supplementary Fig. 2. Correlation between *CAV1* expression and PAM50 ROR

PAM50 ROR category by of tertiles of *CAV1* gene expression among all patients in SCAN-B **(A)** and METABRIC **(B)**. Pearson correlations of *CAV1* gene expression and the 50 individual genes that are part of the PAM50 ROR classification among all patients in SCAN-B **(C)** and METABRIC **(D)**.

Supplementary Fig. 3. Multivariable survival analyses of *CAV1* expression

Forest plots of mutually adjusted hazard ratios (95% confidence intervals) of *CAV1* expression (in tertiles) and other clinicopathologic factors among all patients in relation to recurrence-free interval in SCAN-B (A) and METABRIC (B), distant metastasis-free interval in SCAN-B (C) and METABRIC (D), overall survival in SCAN-B (E) and METABRIC (F), and breast cancer-specific survival (G). The number of patients is indicated at each time-point.

Supplementary Fig. 4. Survival analyses of *CAV1* expression in relation RFI stratified by ROR category

Kaplan-Meier estimates of *CAV1* expression (in tertiles) among ROR High tumors in relation to recurrence-free interval in SCAN-B (**A**) and METABRIC (**B**) and corresponding forest plots of mutually adjusted hazard ratios (95% confidence intervals) in SCAN-B (**C**) and METABRIC (**D**). Kaplan-Meier estimates of *CAV1* expression (in tertiles) among ROR Low/Intermediate tumors in relation to recurrence-free interval in SCAN-B (**E**) and METABRIC (**F**) and corresponding forest plots of mutually adjusted hazard ratios (95% confidence intervals) in SCAN-B (**G**) and METABRIC (**H**). The number of patients is indicated at each time-point.

Supplementary Fig. 5. Survival analyses of *CAV1* expression in relation DMFI stratified by ROR category

Kaplan-Meier estimates of *CAV1* expression (in tertiles) among ROR High tumors in relation to distant metastasis-free interval in SCAN-B (**A**) and METABRIC (**B**) and corresponding forest plots of mutually adjusted hazard ratios (95% confidence intervals) in SCAN-B (**C**) and METABRIC (**D**). Kaplan-Meier estimates of *CAV1* expression (in tertiles) among ROR Low/Intermediate tumors in relation to distant metastasis-free interval in SCAN-B (**E**) and METABRIC (**F**) and corresponding forest plots of mutually adjusted hazard ratios (95% confidence intervals) in SCAN-B (**G**) and METABRIC (**H**). The number of patients is indicated at each time-point.

Supplementary Fig. 6. Survival analyses of *CAV1* expression in relation OS stratified by ROR category

Kaplan-Meier estimates of *CAV1* expression (in tertiles) among ROR High tumors in relation to overall survival in SCAN-B (**A**) and METABRIC (**B**) and corresponding forest plots of mutually adjusted hazard ratios (95% confidence intervals) in SCAN-B (**C**) and METABRIC (**D**). Kaplan-Meier estimates of *CAV1* expression (in tertiles) among ROR Low/Intermediate tumors in relation to overall survival in SCAN-B (**E**) and METABRIC (**F**) and corresponding forest plots of mutually adjusted hazard ratios (95% confidence intervals) in SCAN-B (**G**) and METABRIC (**H**). The number of patients is indicated at each time-point.

Supplementary Fig. 7. Survival analyses of *CAV1* expression in relation BCSS stratified by ROR category

Kaplan-Meier estimates of *CAV1* expression (in tertiles) in METABRIC in relation to breast cancer-specific survival in ROR High tumors (**A**) and ROR Low/Intermediate tumors (**B**) and corresponding forest plots of mutually adjusted hazard ratios (95% confidence intervals) in ROR High tumors (**C**) and ROR Low/Intermediate tumors (**D**). The number of patients is indicated at each time-point.

Supplementary Fig. 8. Survival analyses of ROR category in relation DMFI among patients *CAV1* T1 tumors

Kaplan-Meier estimates of ROR category among *CAV1* T1 tumors in relation to distant metastasis-free interval in SCAN-B (**A**) and METABRIC (**B**) and corresponding forest plots of mutually adjusted hazard ratios (95% confidence intervals) in SCAN-B (**C**) and METABRIC (**D**)The number of patients is indicated at each time-point.

Supplementary Fig. 9. Survival analyses of ROR category in relation DMFI among patients *CAV1* T2 tumors

Kaplan-Meier estimates of ROR category among *CAV1* T2 tumors in relation to distant metastasis-free interval in SCAN-B (**A**) and METABRIC (**B**) and corresponding forest plots of mutually adjusted hazard ratios (95% confidence intervals) in SCAN-B (**C**) and METABRIC (**D**)The number of patients is indicated at each time-point.

Supplementary Fig. 10. Survival analyses of ROR category in relation DMFI among patients *CAV1* T3 tumors

Kaplan-Meier estimates of ROR category among *CAV1* T3 tumors in relation to distant metastasis-free interval in SCAN-B (**A**) and METABRIC (**B**) and corresponding forest plots of mutually adjusted hazard ratios (95% confidence intervals) in SCAN-B (**C**) and METABRIC (**D**)The number of patients is indicated at each time-point.

Supplementary Table 1. Full report of interactions between ROR High and *CAV1* T3 on RFI

**Interaction between ROR High and *CAV1* T3 on RFI – SCAN-B**			
	CAV1 T1	*CAV1* T3	Effect of *CAV1* T3 within each stratum of ROR
	HR (95% CI)	HR (95% CI)	HR (95% CI)
ROR Low/Intermediate	1<br>Reference	1.01 (0.60, 1.70)<br>*P* > 0.3	1.01 (0.60, 1.70)<br>*P* > 0.3
ROR High	1.69 (1.00, 2.85)<br>*P* = 0.048	2.74 (1.58, 4.73)<br>*P* < 0.001	1.62 (1.15, 2.27)<br>*P* = 0.006
Effect of ROR High within each stratum of *CAV1*	1.69 (1.00, 2.85)<br>*P* = 0.048	2.70 (1.79, 4.08)<br>*P* < 0.001	
Multiplicative scale	1.60 (0.88, 2.89)<br>*P* = 0.12		
RERI	1.03 (0.19, 1.87)<br>*P* = 0.008		
AP	0.38 (0.11, 0.65)<br>*P* = 0.003		
**Interaction between ROR High and *CAV1* T3 on RFI – METABRIC**			
	CAV1 T1	*CAV1* T3	Effect of *CAV1* T3 within each stratum of ROR
	HR (95% CI)	HR (95% CI)	HR (95% CI)
ROR Low/Intermediate	1<br>Reference	0.63 (0.42, 0.95)<br>*P* = 0.027	0.63 (0.42, 0.95)<br>*P* = 0.027
ROR High	1.01 (0.69, 1.48)<br>*P* > 0.3	1.01 (0.69, 1.48)<br>*P* > 0.3	0.99 (0.77, 1.28)<br>*P* > 0.3
Effect of ROR High within each stratum of *CAV1*	1.01 (0.69, 1.48)<br>*P* > 0.3	1.59 (1.10, 2.3)<br>*P* = 0.014	
Multiplicative scale	1.57 (0.99, 2.51)<br>*P* = 0.057		
RERI	0.36 (0.01, 0.72)<br>*P* = 0.022		
AP	0.36 (-0.04, 0.76)<br>*P* = 0.038		

RERI – Relative risk due to interaction
AP – Attributable Portion

Supplementary Table 2. Full report of interactions between ROR High and *CAV1* T3 on OS

**Interaction between ROR High and *CAV1* T3 on OS – SCAN-B**			
	CAV1 T1	*CAV1* T3	Effect of *CAV1* T3 within each stratum of ROR
	HR (95% CI)	HR (95% CI)	HR (95% CI)
ROR Low/Intermediate	1<br>Reference	0.80 (0.59, 1.08)<br>*P* = 0.15	0.80 (0.59, 1.08)<br>*P* = 0.15
ROR High	1.15 (0.83, 1.60)<br>*P* > 0.3	1.32 (0.91, 1.91)<br>*P* = 0.15	1.14 (0.85, 1.53)<br>*P* > 0.3
Effect of ROR High within each stratum of *CAV1*	1.15 (0.83, 1.60)<br>*P* > 0.3	1.65 (1.19, 2.28)<br>*P* = 0.003	
Multiplicative scale	1.43 (0.95, 2.14)<br>*P* = 0.084		
RERI	0.36 (-0.05, 0.78)<br>*P* = 0.041		
AP	0.28 (-0.03, 0.58)<br>*P* = 0.036		
**Interaction between ROR High and *CAV1* T3 on OS – METABRIC**			
	CAV1 T1	*CAV1* T3	Effect of *CAV1* T3 within each stratum of ROR
	HR (95% CI)	HR (95% CI)	HR (95% CI)
ROR Low/Intermediate	1<br>Reference	0.63 (0.44, 0.89)<br>*P* = 0.008	0.63 (0.44, 0.89)<br>*P* = 0.008
ROR High	0.89 (0.64, 1.24)<br>*P* > 0.3	1.03 (0.72, 1.46)<br>*P* > 0.3	1.15 (0.92, 1.45)<br>*P* = 0.22
Effect of ROR High within each stratum of *CAV1*	0.89 (0.64, 1.24)<br>*P* > 0.3	1.64 (1.19, 2.26)<br>*P* = 0.003	
Multiplicative scale	1.84 (1.23, 2.75)<br>*P* = 0.003		
RERI	0.51 (0.22, 0.8)<br>*P* < 0.001		
AP	0.49 (0.17, 0.82)<br>*P* = 0.001		

RERI – Relative risk due to interaction

AP – Attributable Portion

Supplementary Table 3. Full report of interactions between ROR High and *CAV1* T3 on BCSS

**Interaction between ROR High and *CAV1* T3 on BCSS – METABRIC**			
	CAV1 T1	*CAV1* T3	Effect of *CAV1* T3 within each stratum of ROR
	HR (95% CI)	HR (95% CI)	HR (95% CI)
ROR Low/Intermediate	1<br>Reference	0.51 (0.31, 0.82)<br>*P* = 0.006	0.51 (0.31, 0.82)<br>*P* = 0.006
ROR High	1.00 (0.65, 1.54)<br>*P* > 0.3	1.28 (0.82, 2.01)<br>*P* = 0.28	1.28 (0.99, 1.66)<br>*P* = 0.060
Effect of ROR High within each stratum of *CAV1*	1.00 (0.65, 1.54)<br>*P* > 0.3	2.52 (1.67, 3.83)<br>*P* <0.001	
Multiplicative scale	2.52 (1.48, 4.28)<br>*P* = 0.001		
RERI	0.77 (0.43, 1.12)<br>*P* < 0.001		
AP	0.60 (0.26, 0.94)<br>*P* = 0.001		

RERI – Relative risk due to interaction

AP – Attributable Portion

Supplementary Fig. 1.

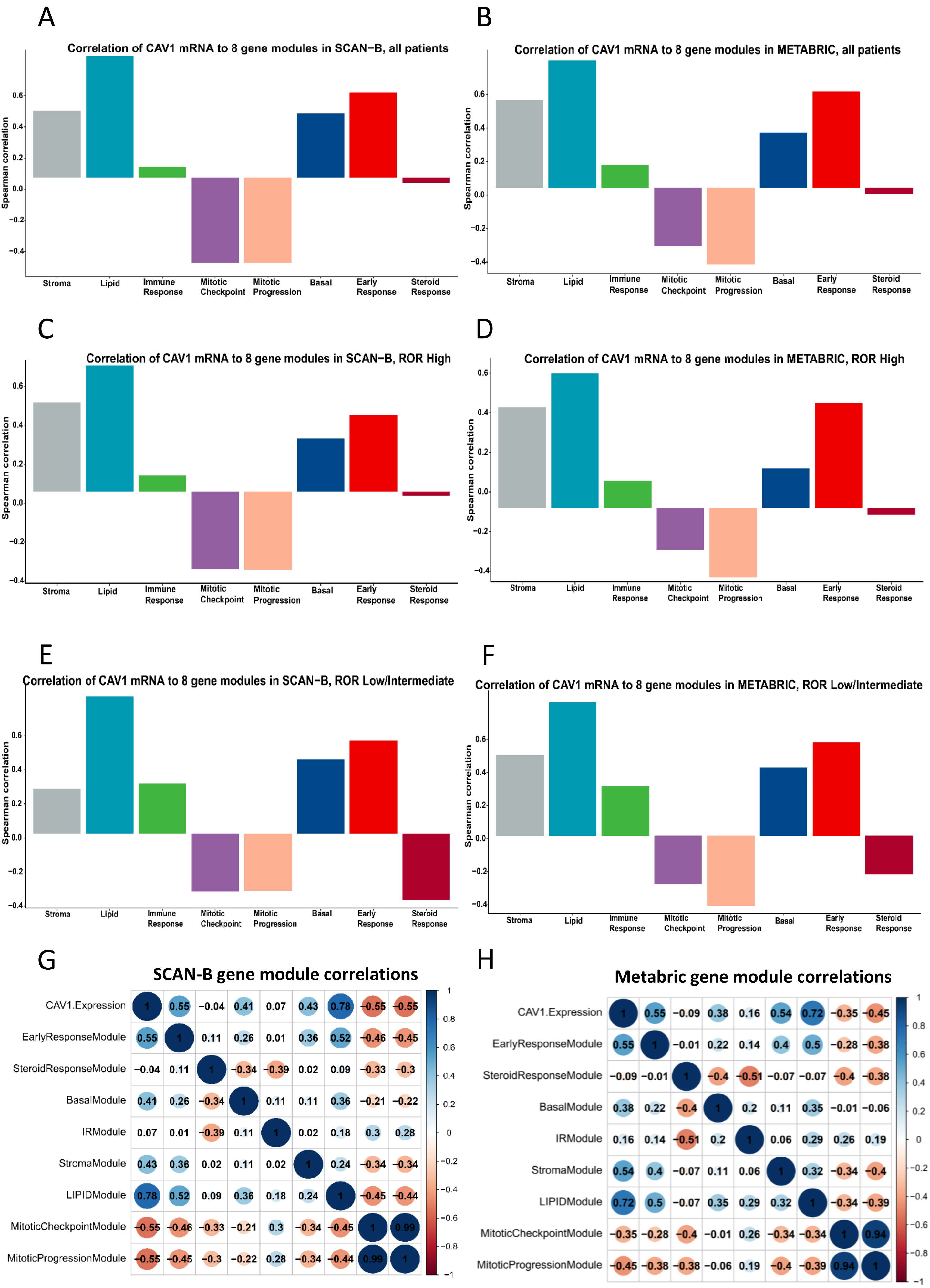

Supplementary Fig. 2.

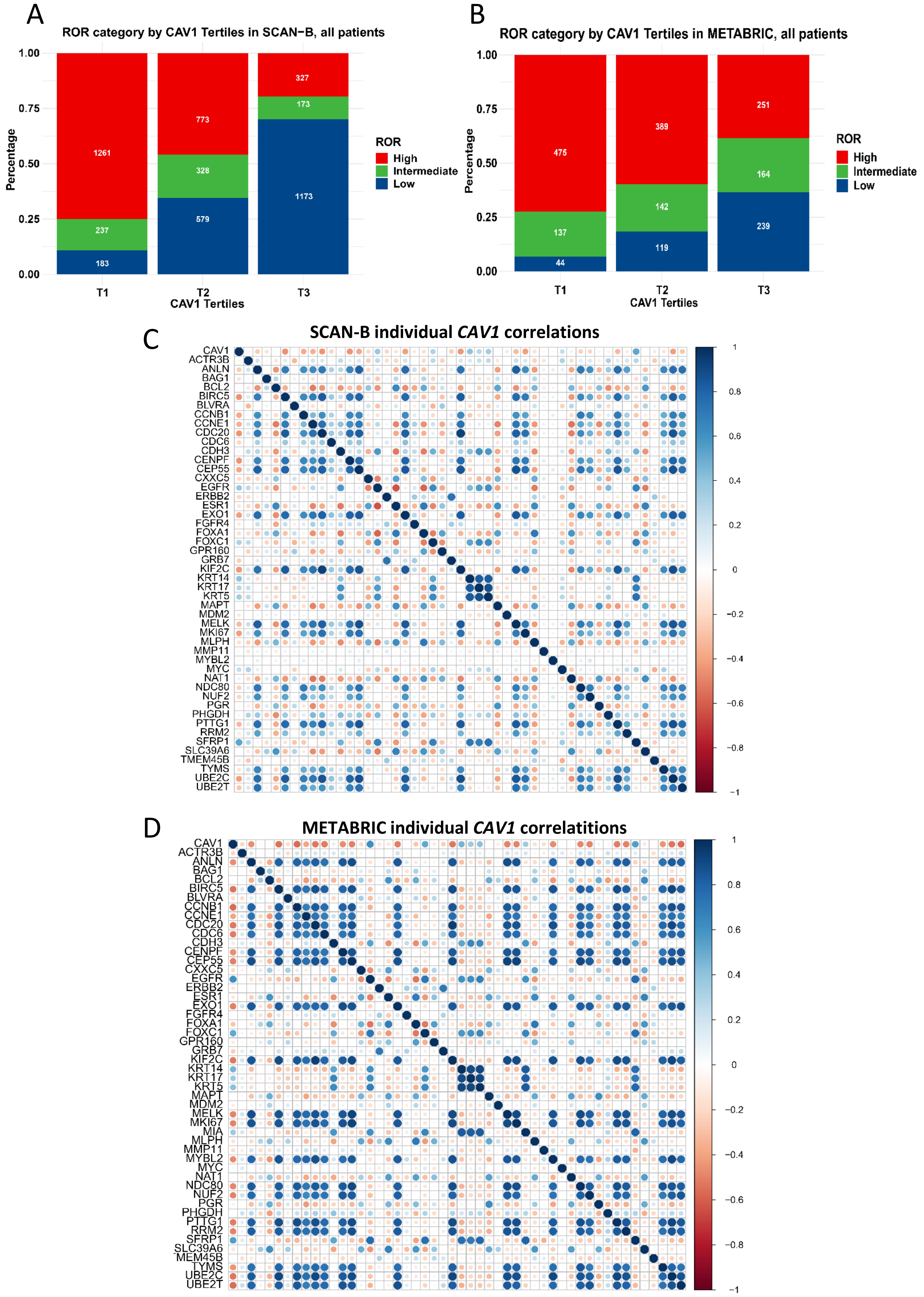

Supplementary Fig. 3.

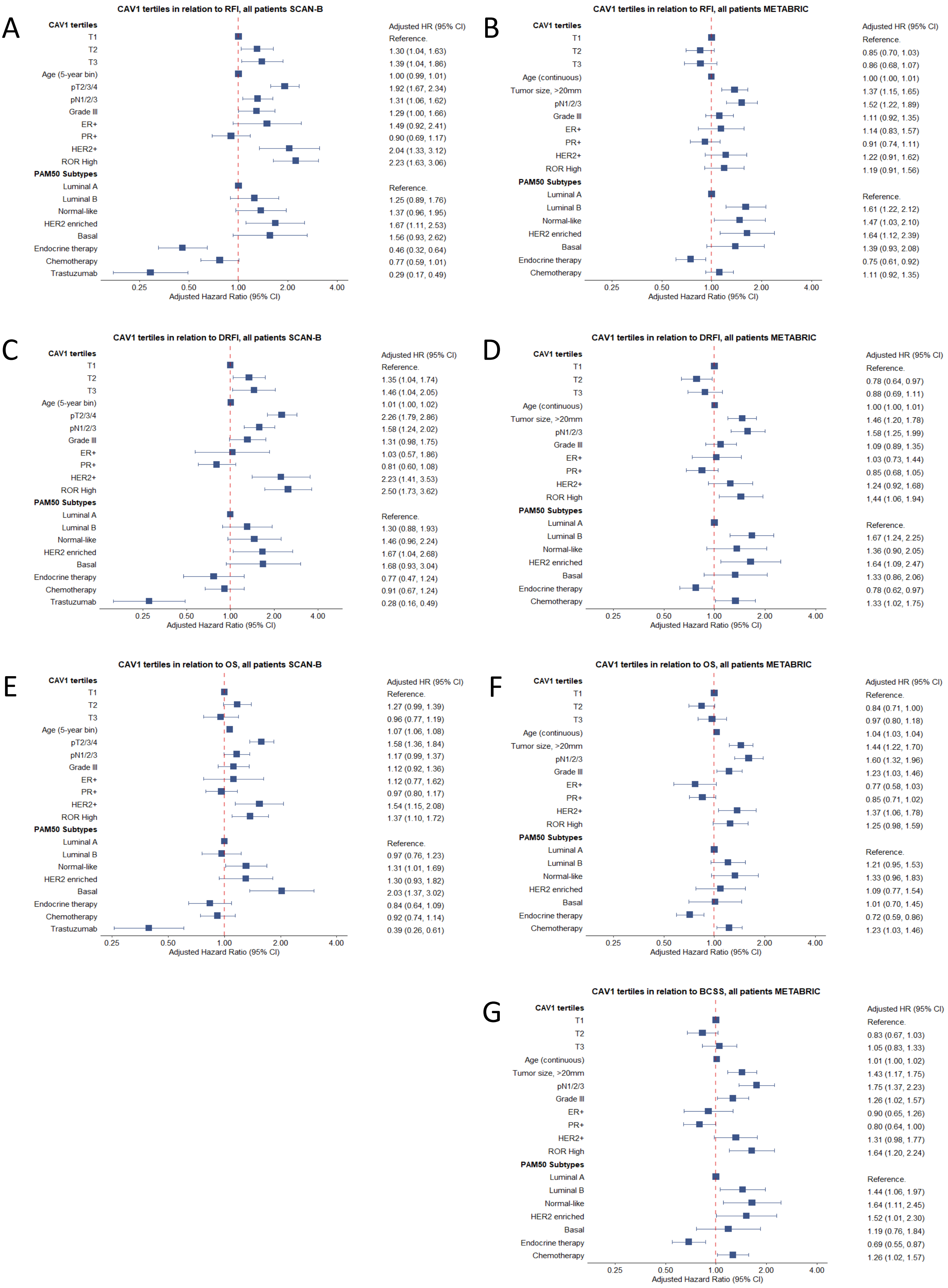

Supplementary Fig. 4.

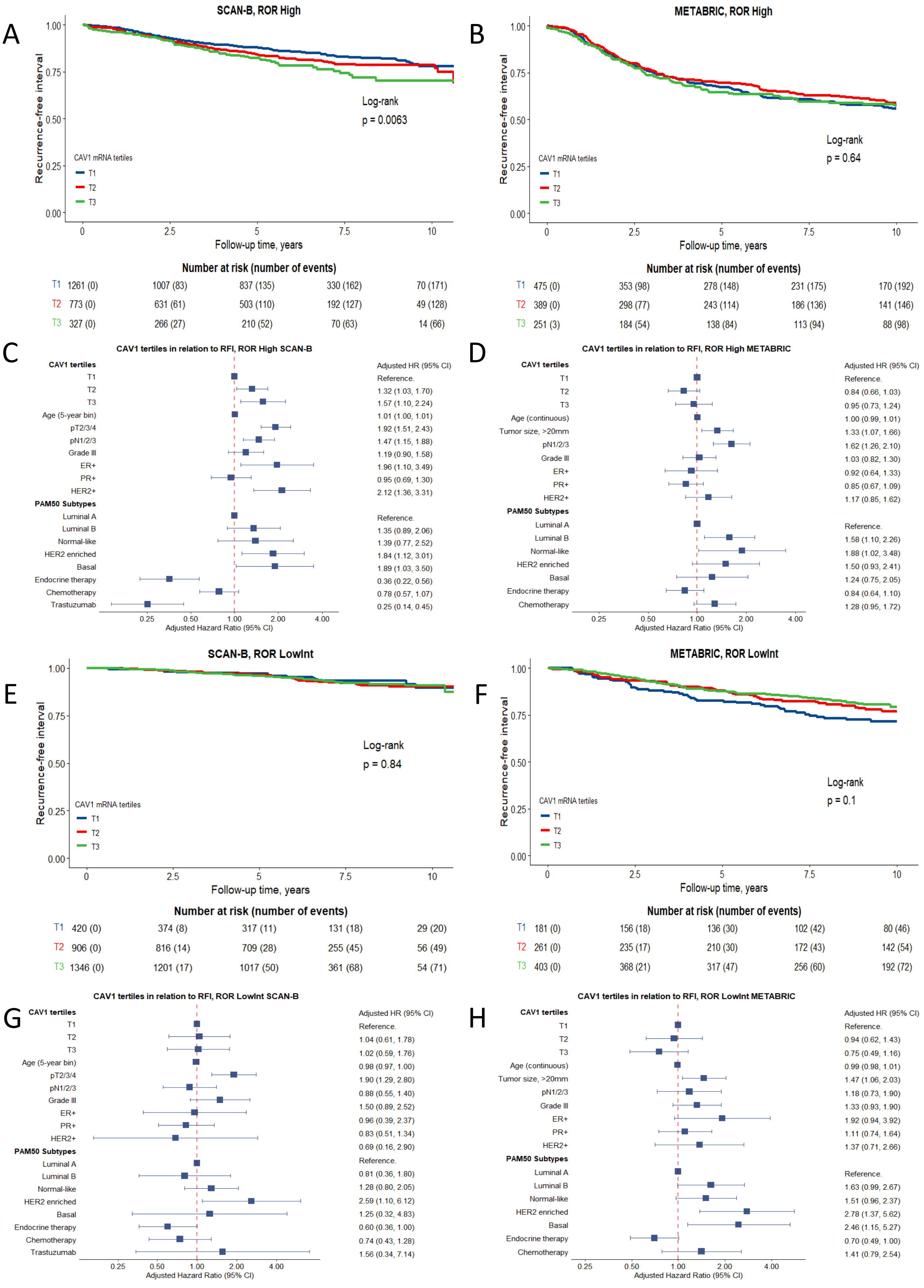

Supplementary Fig. 5.

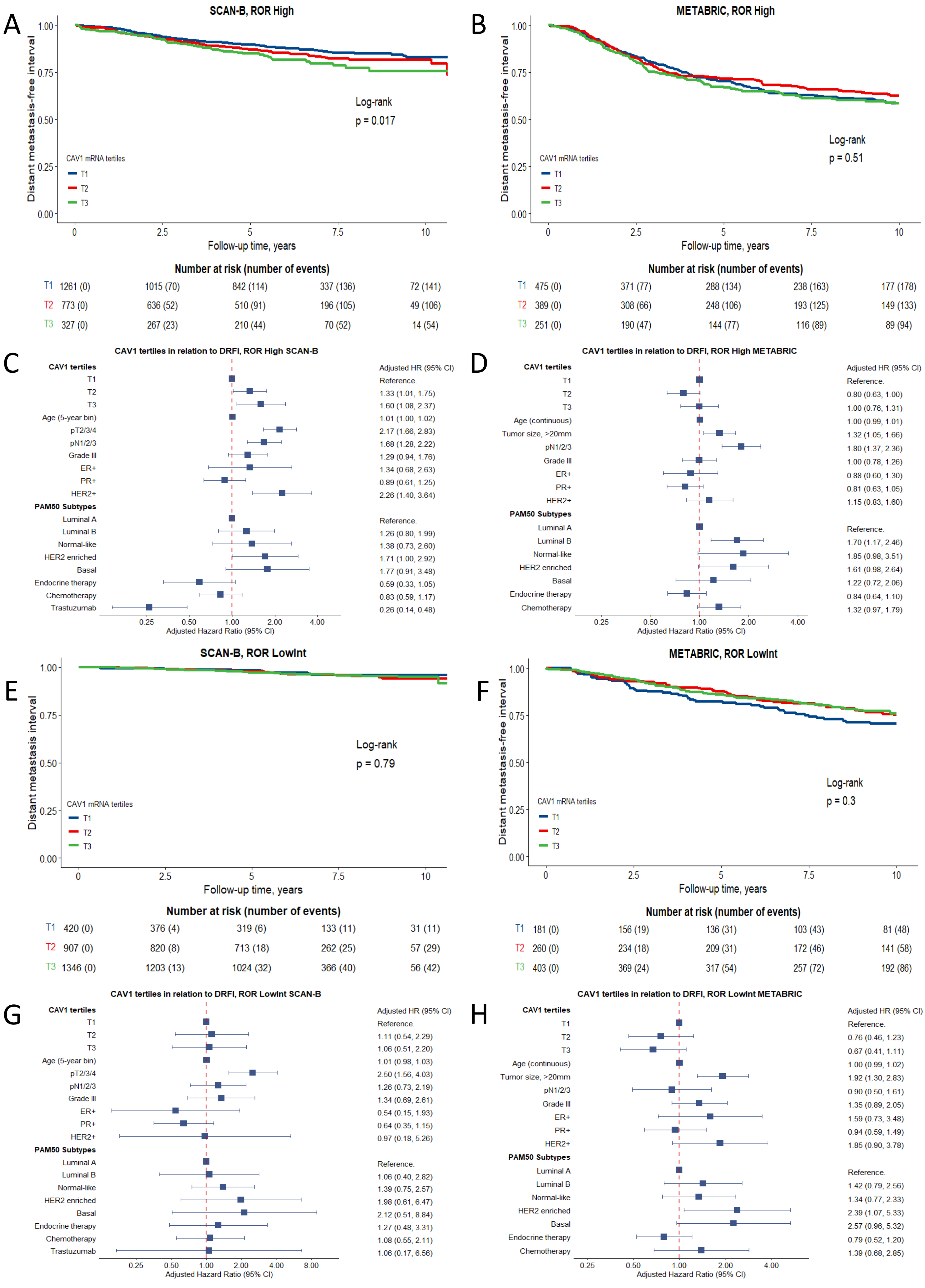

Supplementary Fig. 6.

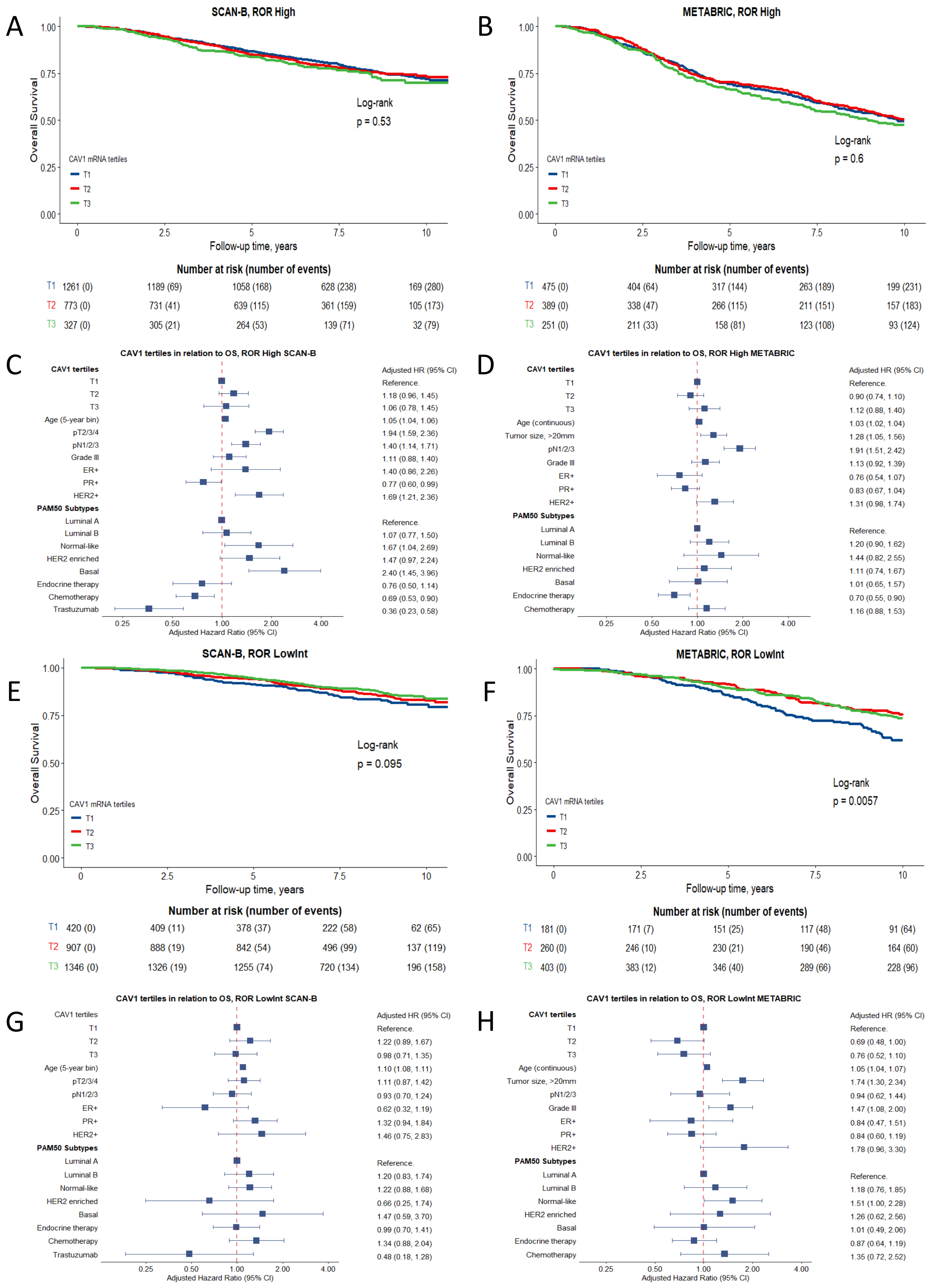

Supplementary Fig. 7.

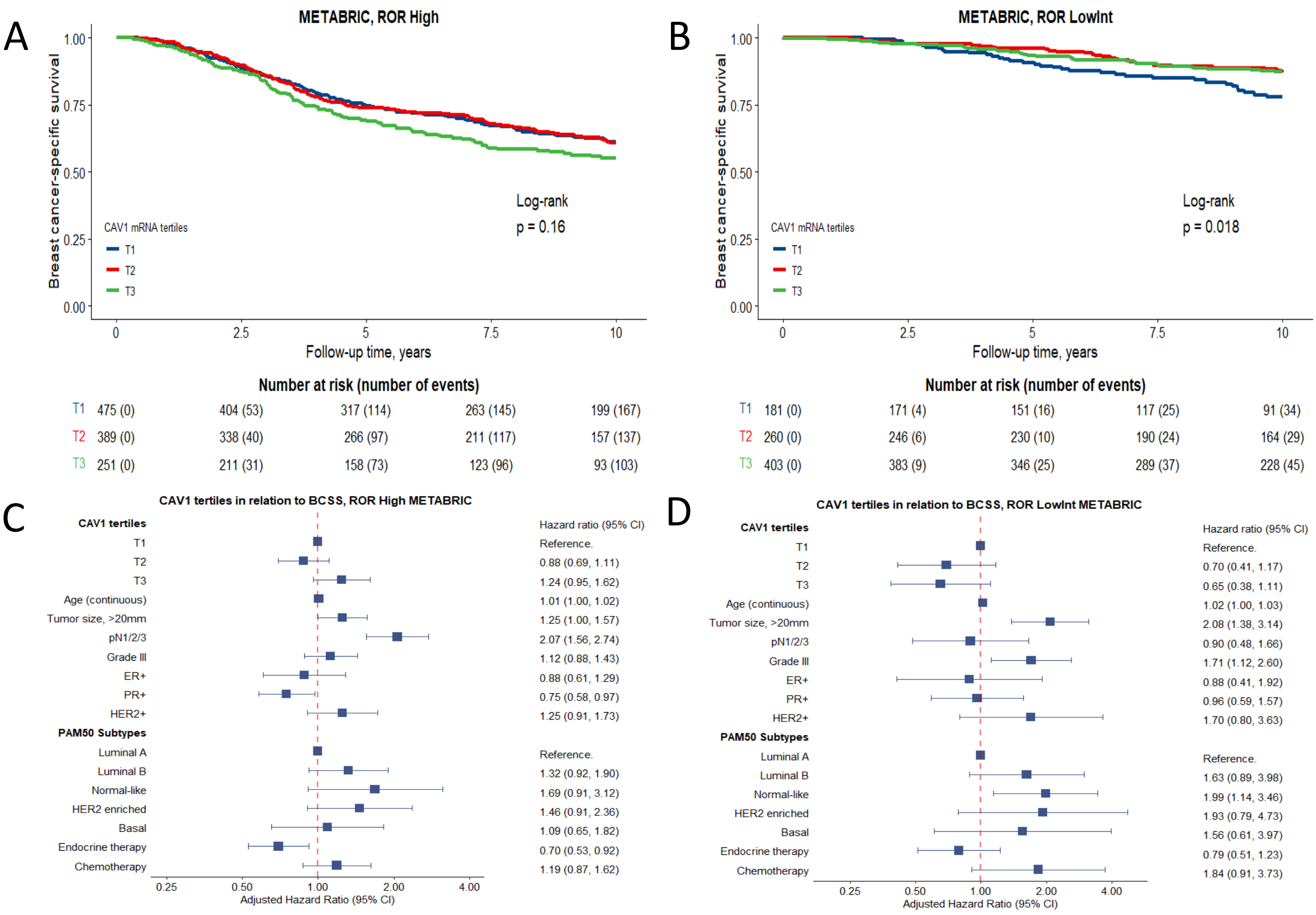

Supplementary Fig. 8.

A

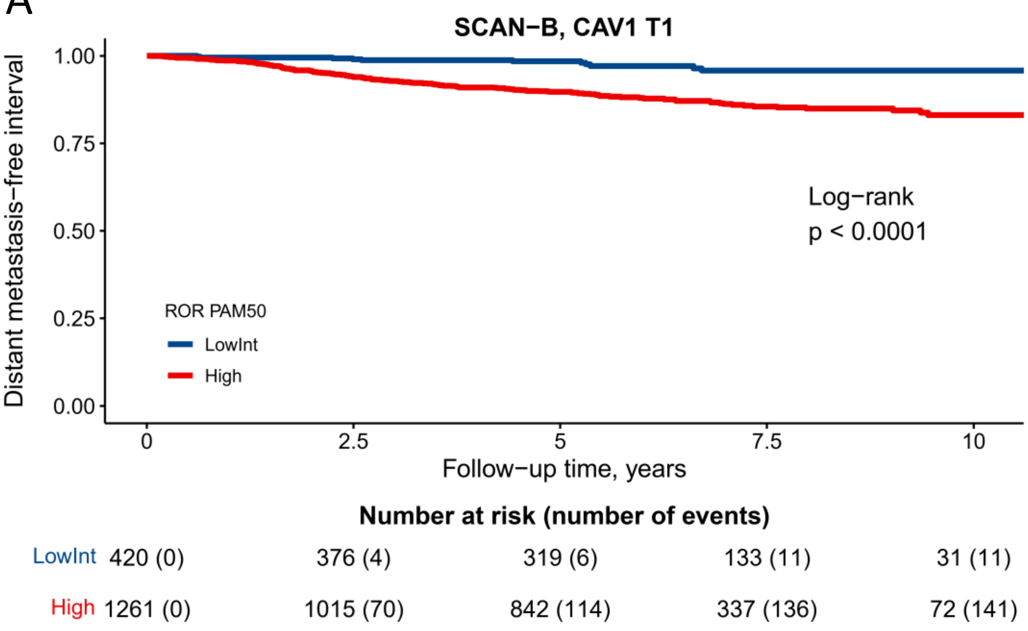

B

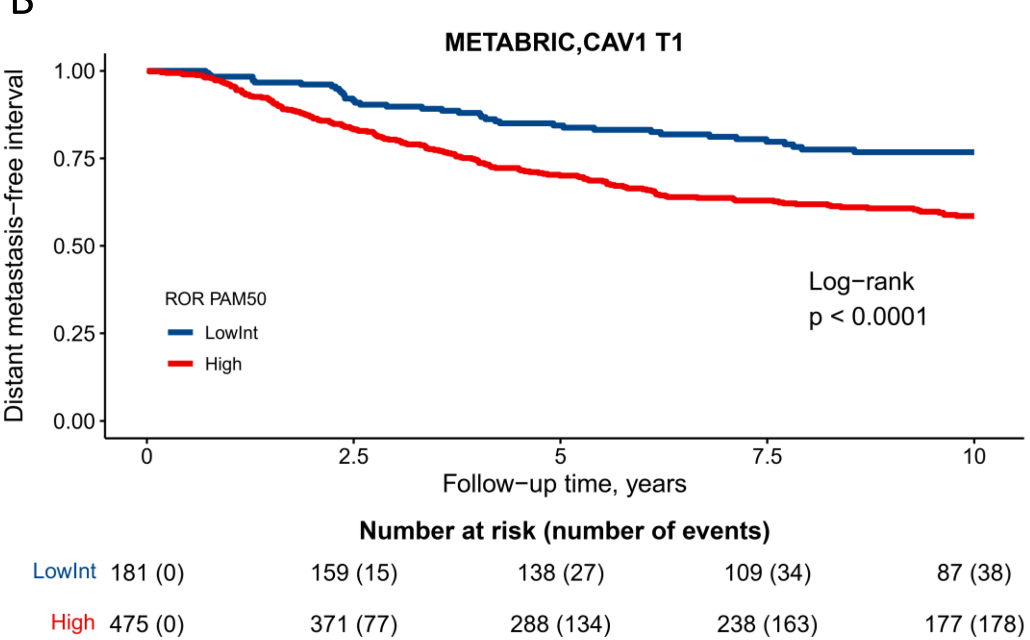

C

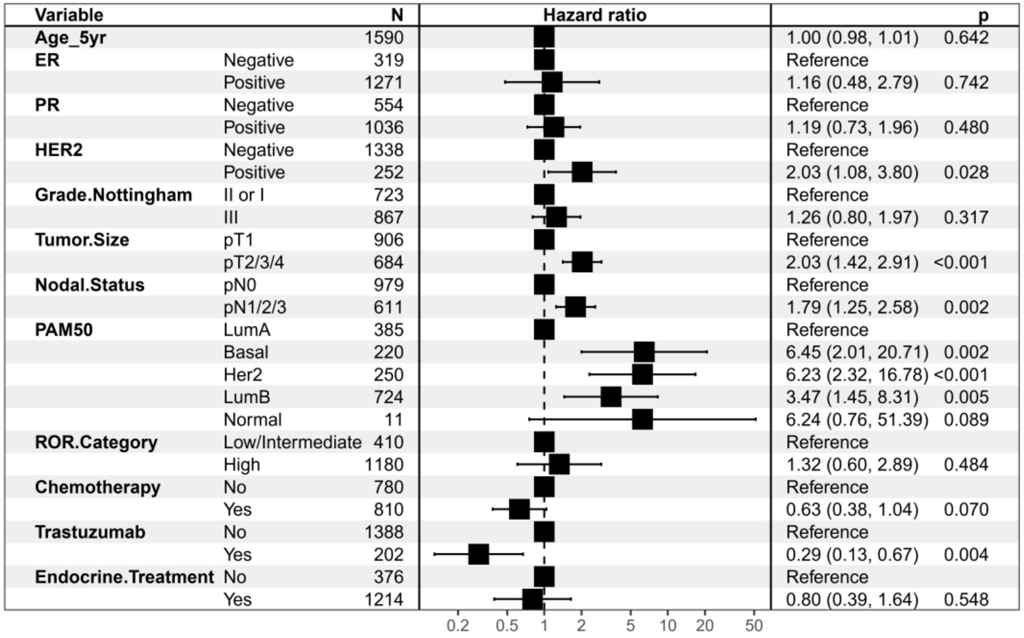

D

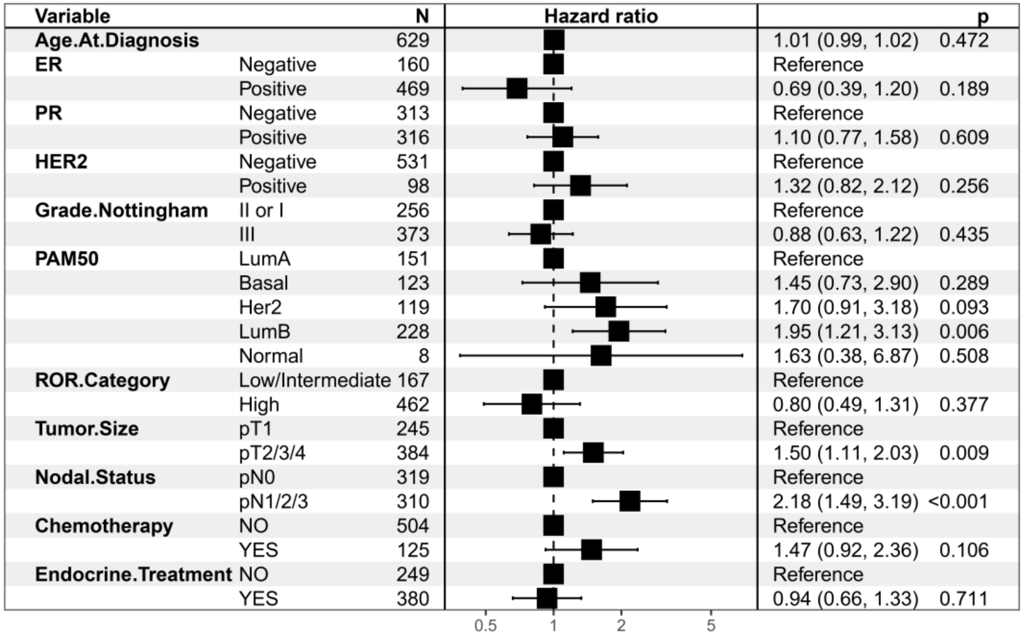

Supplementary Fig. 9.

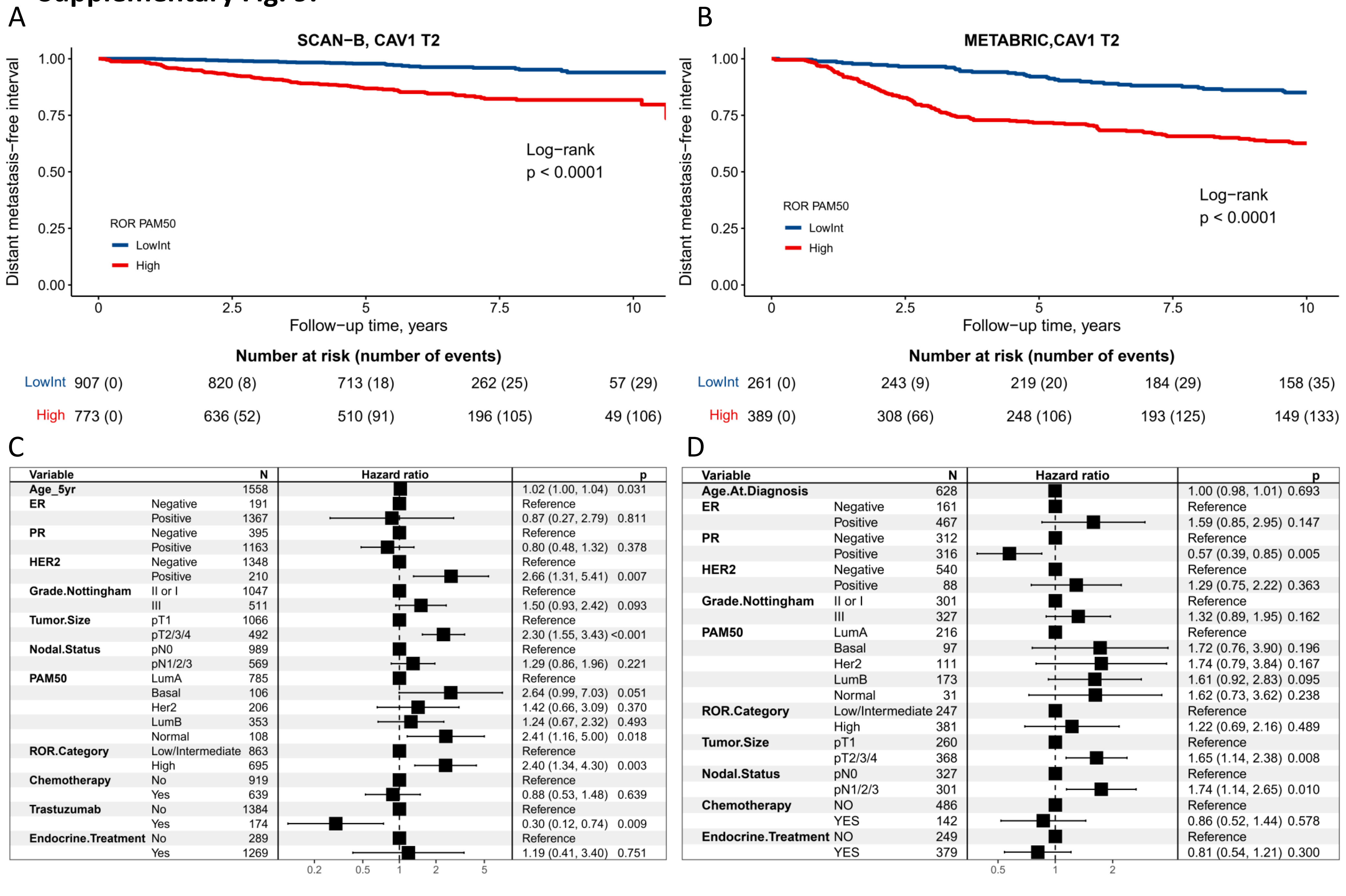

Supplementary Fig. 10.

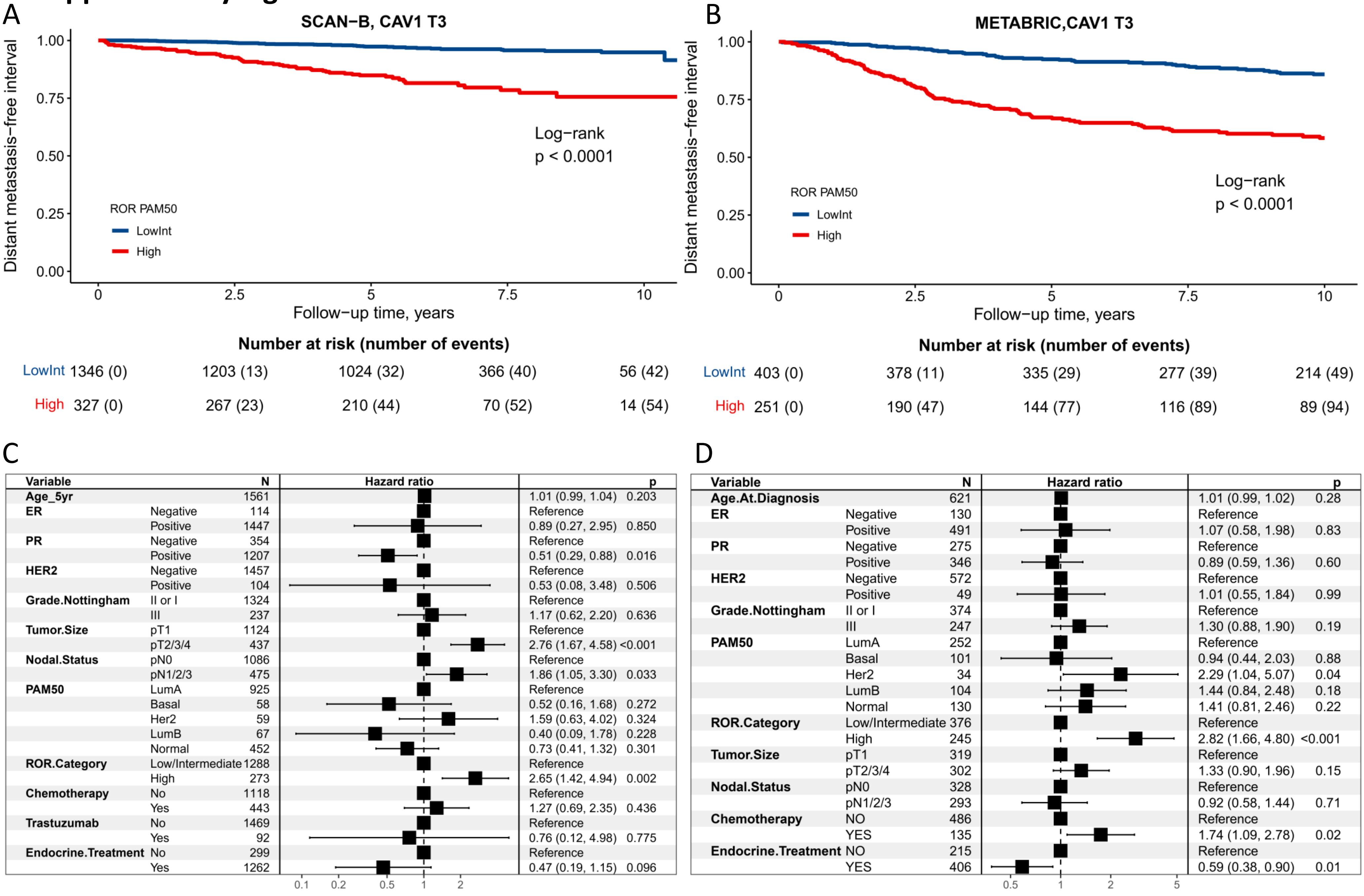

Supplement: Supplementary file 1 — Supplementary Information 1. [file 41598_2024_57365_MOESM1_ESM.pdf]
